# The Development of Tetrazole Derivatives as Protein Arginine Methyltransferase I (PRMT I) Inhibitors

**DOI:** 10.3390/ijms20153840

**Published:** 2019-08-06

**Authors:** Yutong Sun, Zhe Wang, Hao Yang, Xuanli Zhu, Han Wu, Lu Ma, Fang Xu, Wei Hong, Hao Wang

**Affiliations:** 1 School of Pharmacy, Ningxia Medical University, Yinchuan 750004, China; 2 School of Basic Medical Sciences, Ningxia Medical University, Yinchuan 750004, China; 3 School of Postgraduate Education, Ningxia Medical University, Yinchuan 750004, China; 4 School of Chemistry and Chemical Engineering, North Minzu University, Yinchuan 750021, China; 5 Key Laboratory of Chemical Engineering and Technology, State Ethnic Affairs Commission, North Minzu University, Yinchuan 750021, China

**Keywords:** PRMT1, structure-activity relationship (SAR), MOA studies, molecular dynamic simulations, binding mode

## Abstract

Protein arginine methyltransferase 1 (PRMT1) can catalyze protein arginine methylation by transferring the methyl group from *S*-adenosyl-L-methionine (SAM) to the guanidyl nitrogen atom of protein arginine, which influences a variety of biological processes. The dysregulation of PRMT1 is involved in a diverse range of diseases, including cancer. Therefore, there is an urgent need to develop novel and potent PRMT1 inhibitors. In the current manuscript, a series of 1-substituted 1*H*-tetrazole derivatives were designed and synthesized by targeting at the substrate arginine-binding site on PRMT1, and five compounds demonstrated significant inhibitory effects against PRMT1. The most potent PRMT1 inhibitor, compound **9a**, displayed non-competitive pattern with respect to either SAM or substrate arginine, and showed the strong selectivity to PRMT1 compared to PRMT5, which belongs to the type II PRMT family. It was observed that the compound **9a** inhibited the functions of PRMT1 and relative factors within this pathway, and down-regulated the canonical Wnt/β-catenin signaling pathway. The binding of compound **9a** to PRMT1 was carefully analyzed by using molecular dynamic simulations and binding free energy calculations. These studies demonstrate that **9a** was a potent PRMT1 inhibitor, which could be used as lead compound for further drug discovery.

## 1. Introduction

**Figure 1:**
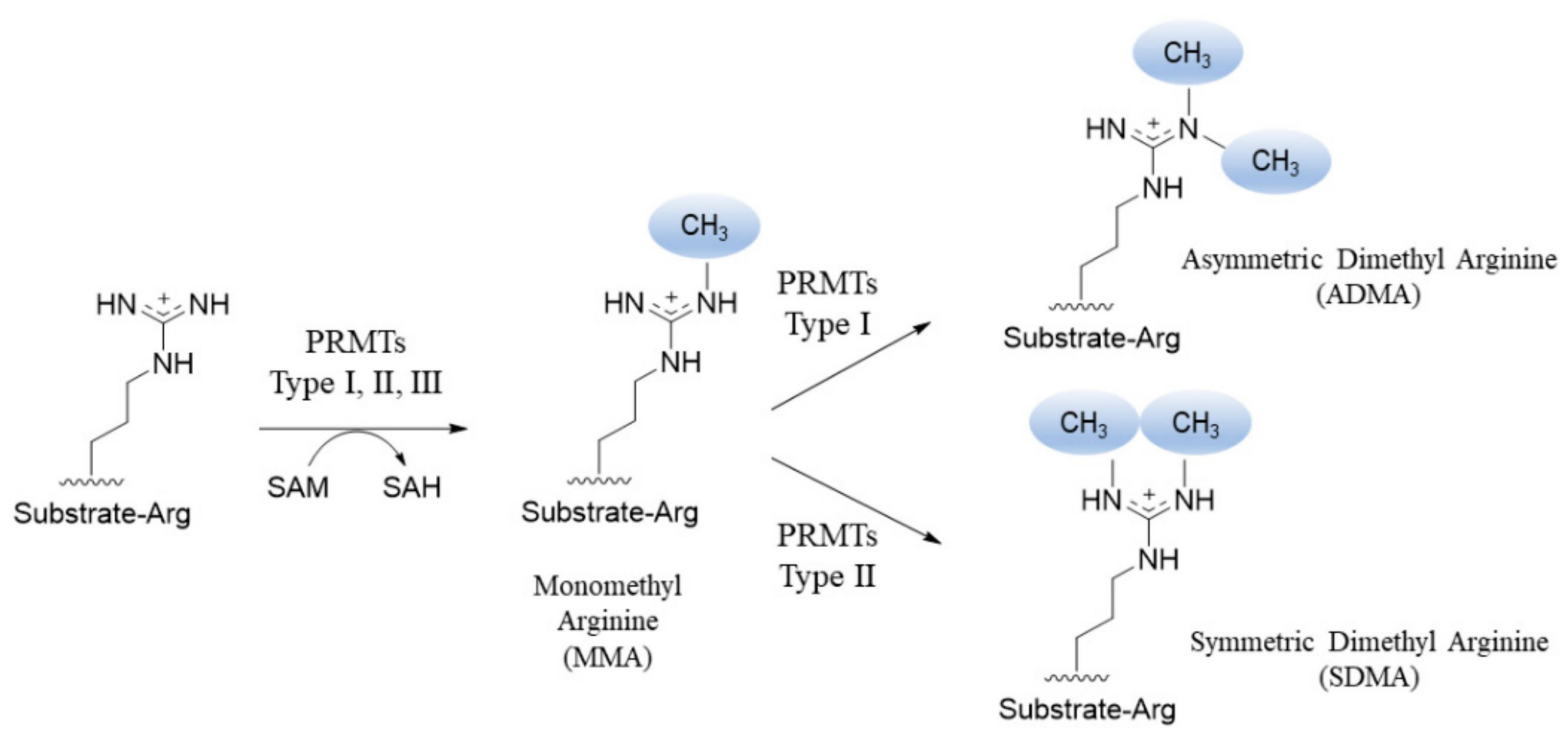
Methylation states of protein arginine through protein arginine methyltransferases (PRMTs).

Protein arginine methyltransferases (PRMTs) transfer the methyl group from *S*-adenosyl-L-methionine (SAM) to the guanidyl nitrogen atom of protein arginine, forming monomethylarginine (mMA) and cofactor *S*-adenosyl-L-homocysteine (SAH) [1,2,3]. Therefore, PRMTs can catalyze protein arginine methylation and influence a wide range of cellular processes, including transcriptional regulation, RNA splicing, cell growth and differentiation [4,5]. According to their different biological characteristics, PRMTs are classified in three categories (Figure 1) [6]. Type І PRMTs (PRMT1, 2, 3, 4, 6 and 8) catalyze mono- and asymmetrical dimethylarginine (ADMA) of arginine residues, whereas, Type II PRMTs (PRMT5 and 9) catalyze mono- and symmetrical dimethylarginine (SDMA) [7]. As the only known Type III PRMT, PRMT7 catalyzes monomethylation of arginine [8].

**Figure 2:**
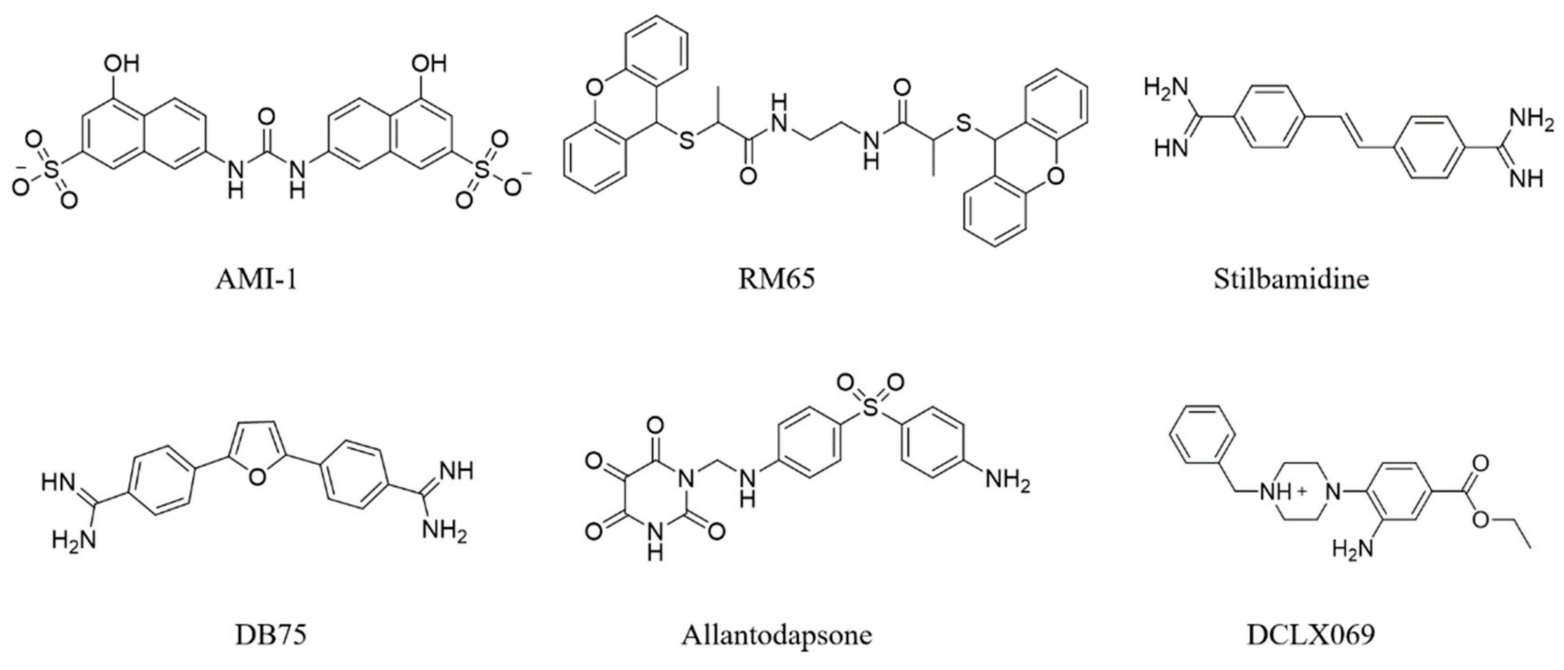
The chemical structures of the typical reported PRMT1 inhibitors.

Previous studies showed that over 90% of the arginine methylation in mammalian cells was produced by PRMT1 catalysis [9]. Furthermore, the disordered expression of PRMT1 is associated with a variety of human diseases including pulmonary fibrosis [10], cardiovascular diseases [11], diabetes, renal diseases [12] and cancer in particular [13,14,15,16]. Overexpression of PRMT1 can promote the growth, survival, metastasis and invasion of tumor cells [17]. Therefore, PRMT1 is considered to be a potential target for the treatment of cancer. Currently, a number of PRMT1 inhibitors have been reported (Figure 2). In 2004, AMI-1 was found through high-throughput screening as the first compound which displayed selectivity to PRMTs over protein lysine methyltransferases (PKMTs) [18]. Later, a number of PRMT1 inhibitors were reported, including RM65 [19], Stilbamidine [20], DB75 [21], Allantodapsone [20] and DCLX069 [22]. However, the weak potency against PRMT1 limited their therapeutic applications. Therefore, there is an urgent need to discover potent PRMT1 inhibitors.

In the current study, a series of 1-substituted 1*H*-tetrazole derivatives were designed and synthesized by targeting at the substrate arginine-binding site on PRMT1, and compound **9a** was discovered to be a potent PRMT1 inhibitor, which contained strong abilities to down-regulate Wnt/β-catenin signaling pathway. Therefore, we suggest that compound **9a** could be an important lead compound for future PRMT-guided drug discovery.

## 2. Results and Discussion

### 2.1. The Molecular Design

**Figure 3:**
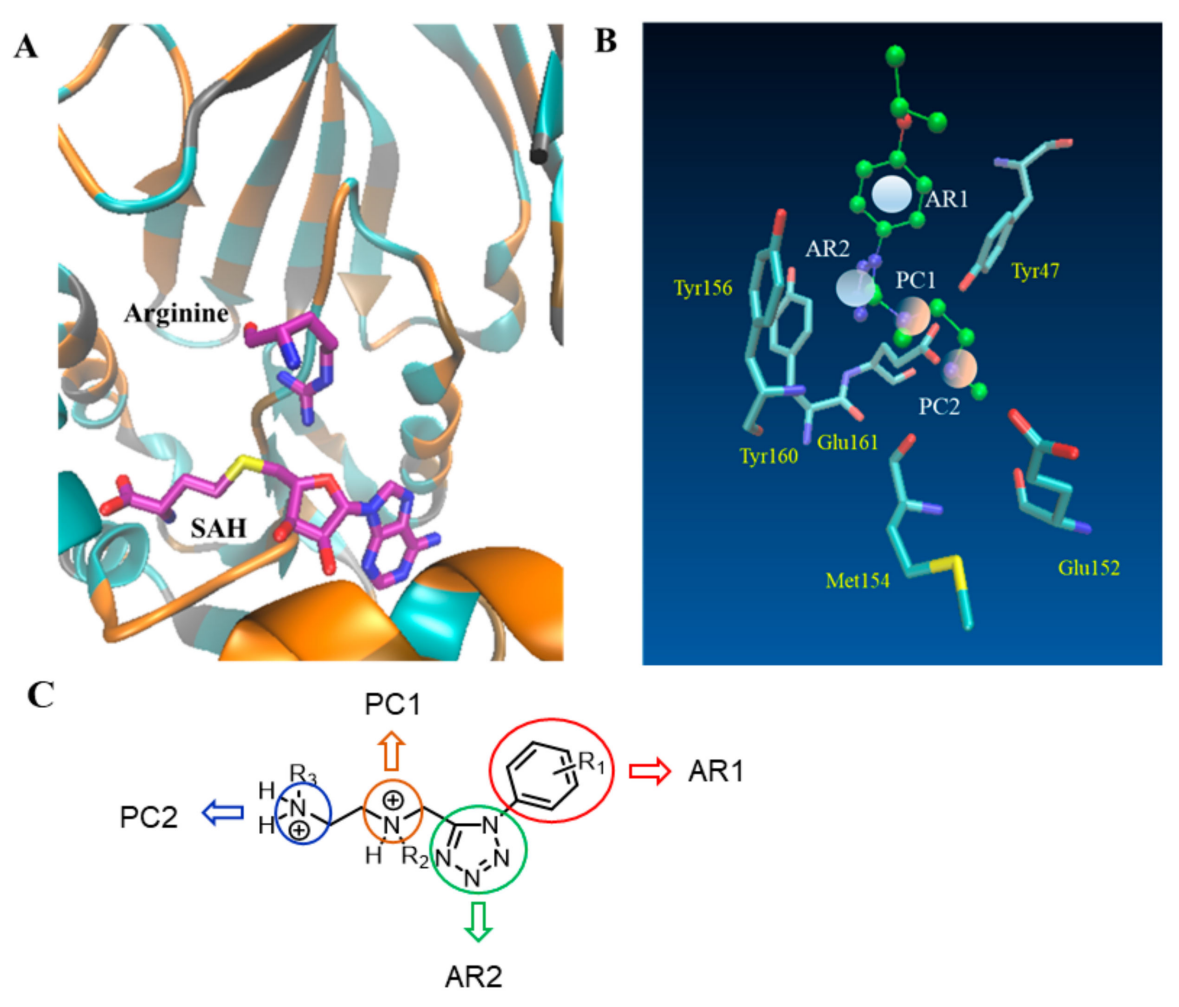
(**A**) The active site of human PRMT1 (hPRMT1) in complex with *S*-adenosyl-L-homocysteine (SAH) and substrate arginine (basic regions are colored in gray, acidic regions are colored in ochre, polar regions are colored in orange and nonpolar regions are colored in cyan); (**B**) The pharmacophores were designed based on the substrate arginine binding site. (AR = aromatic ring and PC = positive center); (**C**) a series of tetrazole derivatives were designed based on the pharmacophores.

The crystal structure of human PRMT1 (hPRMT1) has not yet been solved, so we used our previously published homology model [23] in the current study. As shown in Figure 3A, the active site of PRMT1 is composed of the SAM and substrate arginine binding sites. In theory, if a compound can bind to any sub-site, the methylation will be disturbed. In the current study, we used the arginine binding site as a focus with which to design novel inhibitors. As Figure 3B shows, there are three amino acids with aromatic rings (Tyr47, Tyr156 and Tyr160) that form a hydrophobic pocket, so we designed two aromatic rings to form π-π and hydrophobic interactions with them, which were represented as AR1 and AR2. Two negative charged residues Glu152 and Glu161 were reported to form strong interactions with substrate arginine [24,25], so two positive charged centers were designed, which were represented as PC1 and PC2. In our previous publication [24], a group of compounds with central core of phenyl (AR1) substituted furan (AR2) were discovered as PRMT1 inhibitors. In the current study, we used the tetrazole ring to replace the furan ring as the bioisosteres for designing novel PRMT1 inhibitors. Moreover, in order to investigate the linker length between PC1 and PC2, the different linking groups were incorporated, such as ethyl, propyl, piperazine. According to the pharmacophores and analysis, a series of 1-phenyl substituted tetrazole derivatives (Figure 3C) were designed, in which the benzene and tetrazole ring were according to AR1 and AR2 respectively, and the tertiary amine and secondary amine were according to the PC1 and PC2, respectively.

### 2.2. Chemistry

**Scheme 1:**
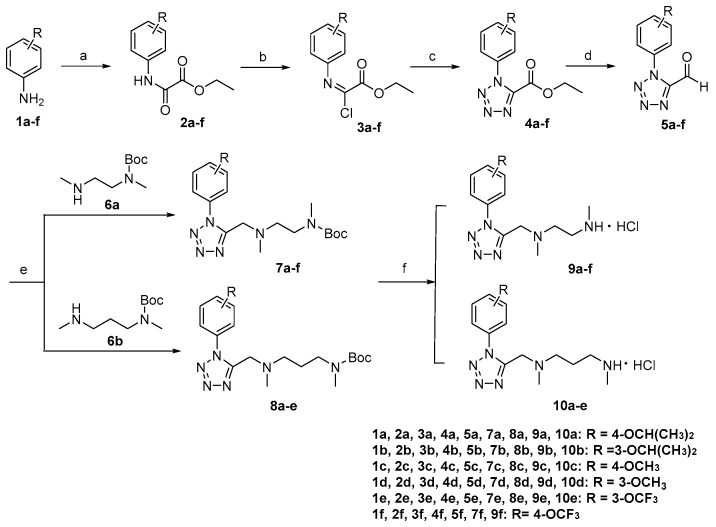
Syntheses of **9a**–**f** and **10a**–**e**. Reagents and conditions: (**a**) ethyl oxalyl monochloride, Et_3_N, CH_2_Cl_2_, RT; (**b**) Ph_3_P, CCl_4_, reflux; (**c**) NaN_3_, CH_3_CN, RT; (**d**) DIBAL-H, CH_2_Cl_2_, −78 °C; (**e**) STAB, DCE, RT; (**f**) HCl/EtOH, RT.

**Scheme 2:**
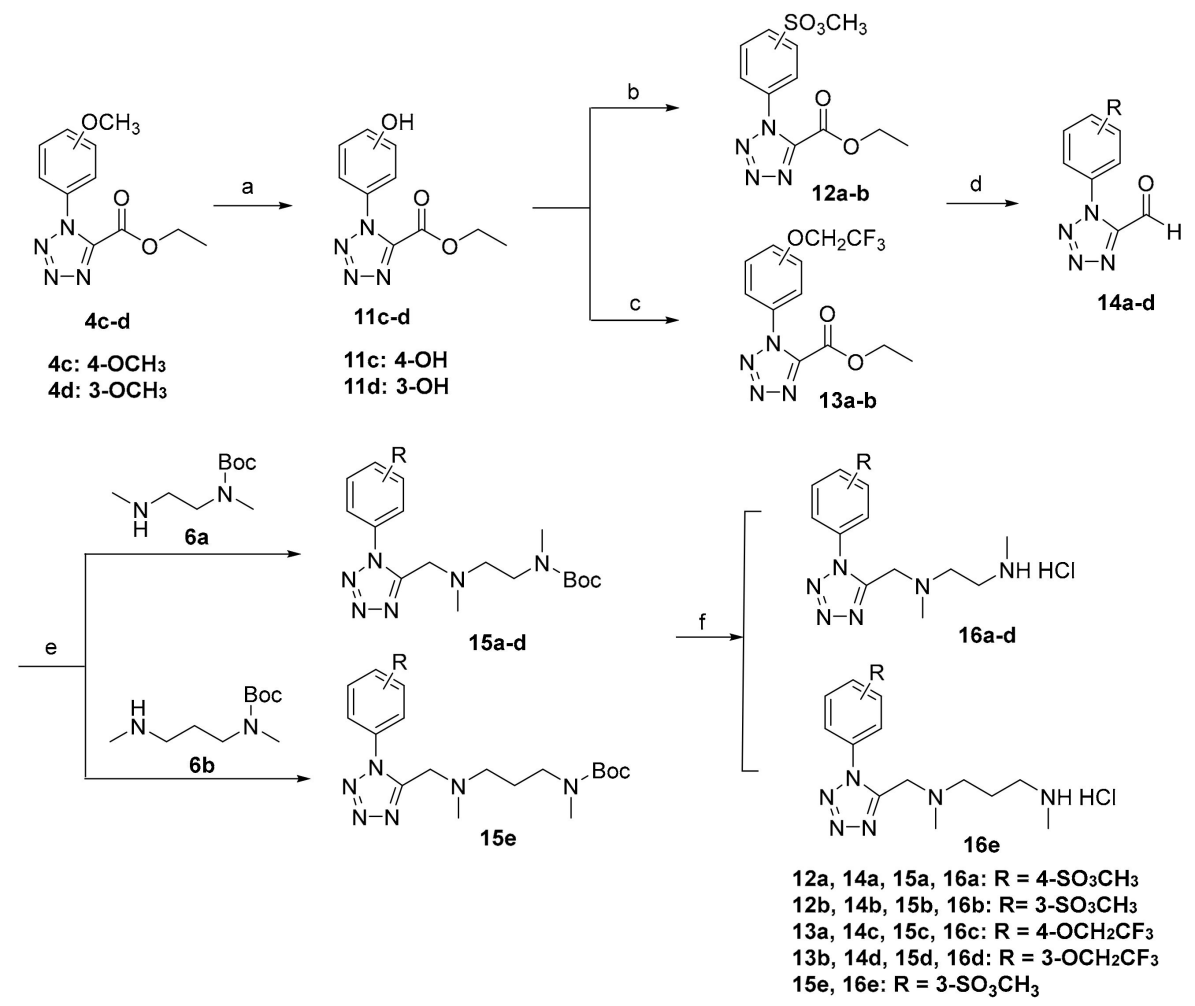
Syntheses of **16a**–**e**. Reagents and conditions: (**a**) BBr_3_, CH_2_Cl_2_, −78°C - RT; (**b**) CH_3_SO_2_Cl, Et_3_N, ethyl acetate, 0 °C; (**c**) CF_3_CH_2_OTf, K_2_CO_3_, DMF, RT; (**d**) DIBAL-H, CH_2_Cl_2_, −78 °C; (**e**) STAB, DCE, RT; (**f**) HCl/EtOH, RT.

**Scheme 3:**
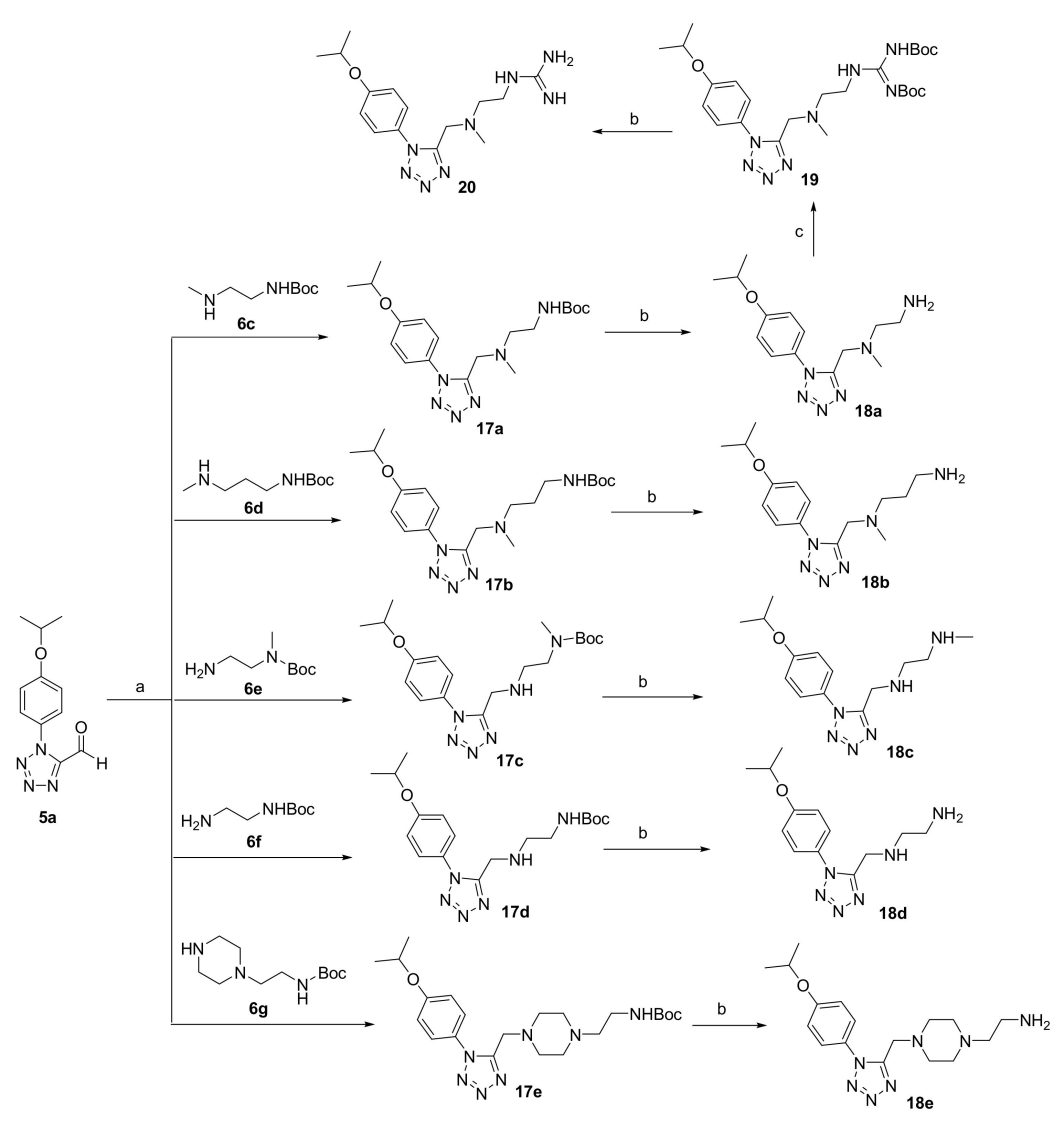
Syntheses of **18a**–**e** and **20**. Reagents and conditions: (**a**) STAB, DCE, RT; (**b**) HCl/EtOH, RT; (**c**) 1,3-bis(tert-butoxycarbonyl)-2-methyl-2-thiopseudourea, Et_3_N, HgCl_2_, DMF, RT.

A series of 1,5-substituded tetrazole derivatives **9a**–**f**, **10a**–**e**, **16a**–**e, 18a**–**e** and **20** were synthesized as illustrated in Scheme 1, Scheme 2 and Scheme 3. In Scheme 1, the commercially available substituted anilines **1a–f** were respectively reacted with ethyl oxalyl monochloride in the presence of triethylamine in anhydrous dichloromethane to generate the compounds **2a**–**f** in good yields (90%–95%). Treatment of **2a**–**f** with triphenylphosphine under refluxing in carbon tetrachloride gave compounds **3a**–**f**, which were reacted directly with sodium azide in acetonitrile to generate 1-substituted phenyl-1*H*-tetrazole-5-carboxylate ethylesters **4a**–**f** in moderate yields over two steps (63%–65%). Then compounds **4a**–**f** reduced by diisobutylaluminium hydride (DIBAL-H) to form 1-substituted phenyl-1*H*-tetrazole-5-aldehydes **5a**–**f** in moderate yields (75%–80%). Meanwhile, the side chains **6a** and **6b** were prepared according the literature protocol [26]. Subsequently, the compounds **5a**–**f** were respectively treated with compound **6a** and **6b** by reductive amination using sodium triacetoxyborohydride (STAB) in dichloroethane to generate compounds **7a**–**f** and **8a**–**e** in moderate yields (75%–85%). Finally, the target compounds **9a**–**f** and **10a**–**e** were obtained by deprotection with saturated hydrochloric acid ethanol solution in good yields (92%–98%).

The syntheses of tetrazole derivatives **16a–e** were depicted in Scheme 2. The compounds **4c–d** could be converted into **11c–d** by demethylation using boron tribromide in the yield of 67%. Then the treatment of compounds **11c–d** with methanesulfonyl chloride in the presence of triethylamine by sulfonylation generated **12a**–**b**. Meanwhile, the compounds **11c**–**d** were reacted with 2,2,2-trifluoroethyltrifluoromethanesulfonate in the presence of potassium carbonate by alkylation to give **13a**–**b**. The intermediate **12a**–**b** and **13a**–**b** were directly reduced by DIBAL-Hto form **14a**–**d** in moderate yields over two steps (75%–78%). Subsequently similar with the procedure in Scheme 1, compounds **14a**–**d** were respectively reacted with side chains **6a** and **6b** by reductive amination to generated compounds **15a–e**, which were deprotected with saturated hydrochloric acid ethanol solution to afford the target compounds **16a–e** in good yields (92%–95%).

The syntheses of tetrazole derivatives **18a**–**e** and **20** were illustrated in Scheme 3. The side chains **6c**–**e** were commercially available, and the preparation of the side chains **6f**–**g** was followed the reported methods [24,27]. Then the compound **5a** was reacted with varied side chains **6c**–**g** by reductive amination to generate the compounds **17a**–**e** in moderate yields (75%–85%). The target compounds **18a**–**e** were obtained by deprotection with saturated hydrochloric acid ethanol solution in good yields (92%–95%). Moreover, the compound **18a** was reacted with 1,3-bis(tert-butoxycarbonyl)-2-methyl-2-thiopseudourea in the presence of triethylamine and mercury dichloride to give compound **19** in the yield of 71%. Finally, the target compound **20** was afforded by deprotection with saturated hydrochloric acid ethanol solution in the yield of 92%.

### 2.3. In vitro PRMT1 Inhibition Assays and Selectivity Assays

A series of 1,5-substituded tetrazole derivatives were synthesized to investigate the structure-activity relationship (SAR) of PRMT1 inhibitors (Table 1). Initially, we designed and synthesized a series of molecules (**9a**–**e, 16a**–**d**, Group I) as shown in Table 1, which contained substituted phenyl on 1*H*-tetrazol and ethylenediamine side chain. The chemical modification of these compounds is mainly focused on the substituted benzene ring. The initial screening of these compounds was carried out using the radioactive PRMT1 methylation inhibition assay, which measured the amount of methyl groups that transferred from [^3^H]-SAM to a biotinylated histone H4 peptide (ac- SGRGKGGKGLGKGGAKRHRKVGGK(Biotin)). In the assay, SAH and AMI-1 were used as the positive controls. It was found that, in Group I, the inhibitory activities of the para-substitution compounds were in general better than those with meta-substitution. Three compounds (**9a**, **9f**, **16c**), which contained 4-OCH(CH_3_)_2_, 4-OCF_3_ and 4-OCH_2_CF_3_ respectively, showed strong inhibitory effects (over 47%) at 10 μM against PRMT1 in the initial screening, and were selected for the IC_50_ determinations, which were 3.5 μM, 23.8 μM and 19.9 μM, respectively. It should be noted that the IC_50_ of compound **9a** is around seven times weaker than SAH but around 20 times stronger than AMI-1 (Table 1). In the second step, the Group II compounds were designed and synthesized by extending the ethylenediamine side chain to propylenediamine. However, the inhibitory activities of all the compounds in the Group II (**10a**–**e**, **16e**) were either totally abolished or remarkably reduced in comparison with those of Group I. In the third step, in order to evaluate the influence of other amino side chains, we designed and synthesized a series of compounds (**18a**–**e**, **20**, Group III) based on the structure of compound **9a**. Among these compounds, **18a** and **18e** exhibited 10.0 μM and 29.0 μM IC_50_s respectively against PRMT1, while the other compounds (**18b**–**d**, **20**) showed relatively poor activities and their inhibitory effects were all lower than 39% at a concentration of 10 μM.

**Figure 4:**
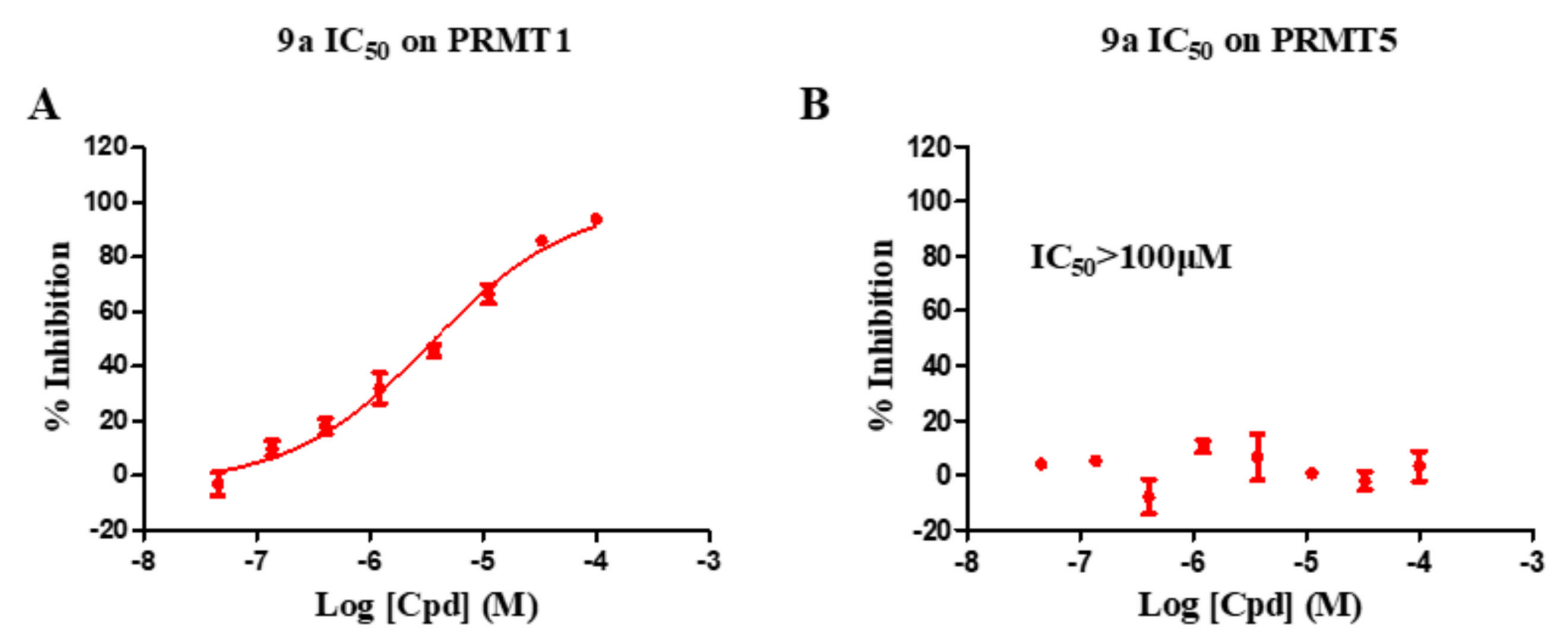
Inhibitory data of Compound **9a** against PRMT1 and PRMT5. The IC_50_ determination for **9a** against the PRMT1 (**A**) and PRMT5 (**B**) based on the radioactive methylation inhibition assay.

To further investigate the selectivity profiles of compounds **9a** (represented in Figure 4), **9f**, **16c**, **18a** and **18e**, the IC_50_s against PRMT5 (the type II PRMT) were measured over 100 μM, as illustrated in Table 1. It suggested that in comparison with type II PRMT, these inhibitors showed good selectivity against PRMT1.

### 2.4. Mechanism of Action (MOA) Study of the Compound **9a**

**Figure 5:**
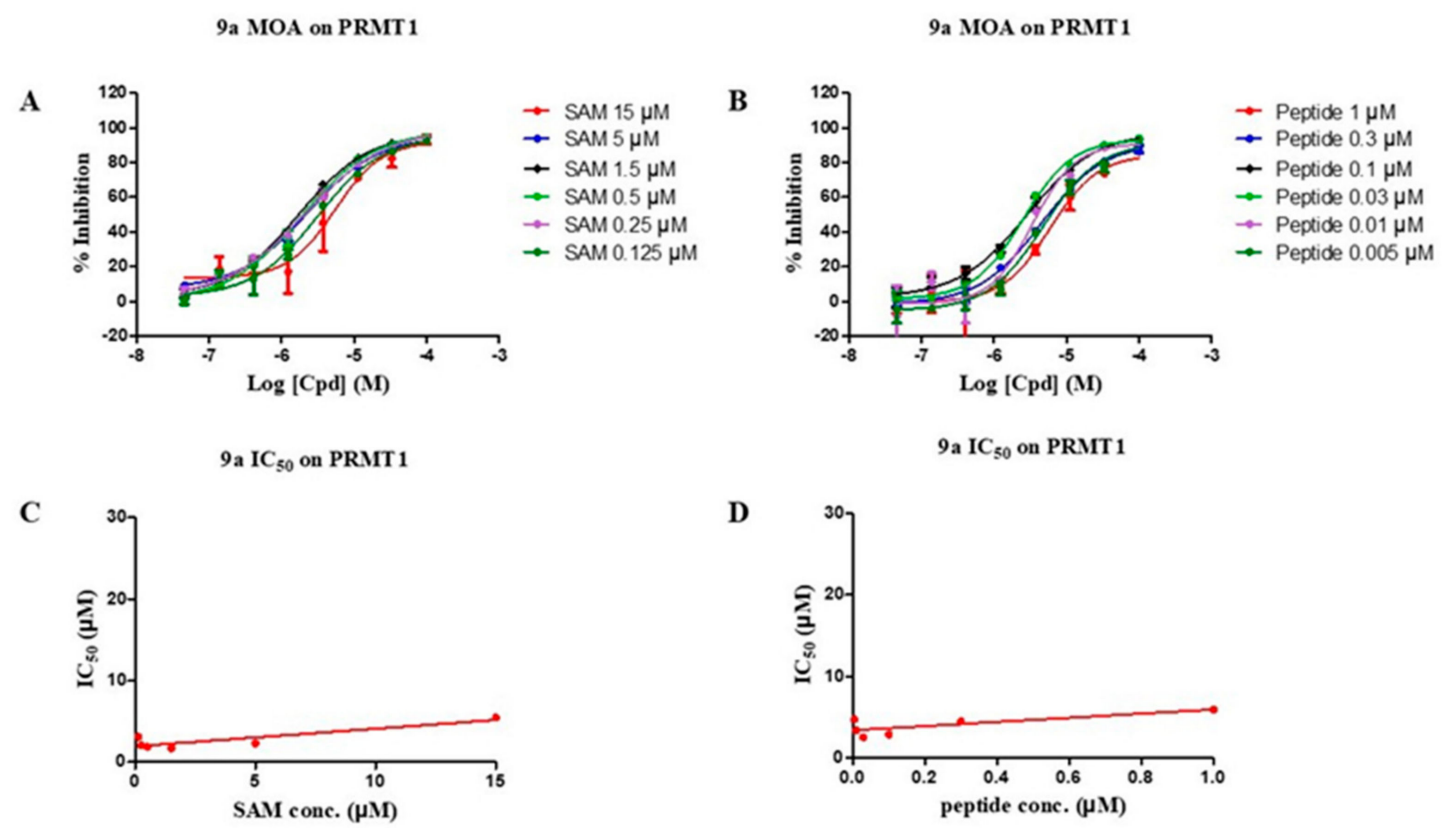
Mechanism of Action (MOA) study results. (**A**,**C**) Competition assay with SAM on PRMT1. (**B**,**D**) Competition assay with Peptide on PRMT1.

The most potent PRMT1 inhibitor in the current study, compound **9a**, was selected for the Mechanism of Action (MOA) study. As illustrated in the Figure 5, various concentrations of SAM or peptide were used to evaluate the potency (IC_50_) of **9a,** and no significant changes were observed according to the concentration changes of either the SAM or peptide. The result of the MOA studies showed that the compound **9a** is a noncompetitive inhibitor for either the cofactor SAM or peptide substrate. Does this result indicate that the compound **9a** inhibit PRMT1 by binding to an allosteric site? Both PRMT1 and PRMT6 are Type I PRMTs, and their structures share the high degree similarity. The co-crystal structure of PRMT6 and its inhibitor, MS023, which also contains ethylenediamine moiety, was solved and clearly showed that ethylenediamine moiety occupied the substrate arginine binding site. Based on the crystal structure, it can be predicated that MS023 should be a competitive inhibitor against the peptide. However, similar to our result, the MOA results showed that the MS023 was noncompetitive with either SAM or peptide substrate [28]. Therefore, we believed that compound **9a** may occupied the substrate arginine binding site, although the MOA results did not show competitive inhibition effects with respect to the peptide. There are potential explanations for the current contradictory results. First, the main binding effects of the substrate were from the outside regions of arginine-binding site. Therefore, compound **9a** did occupy the arginine-binding site, but it did not affect the binding of the whole substrate peptides. Second, the binding of compound **9a** possibly induced major protein conformational changes, which caused the peptides could not enter the binding site to compete with compound **9a** even at the high concentration of the peptides.

### 2.5. Molecular Modeling Study of the Interactions between Compound **9a** and hPRMT1-SAH Complex

**Figure 6:**
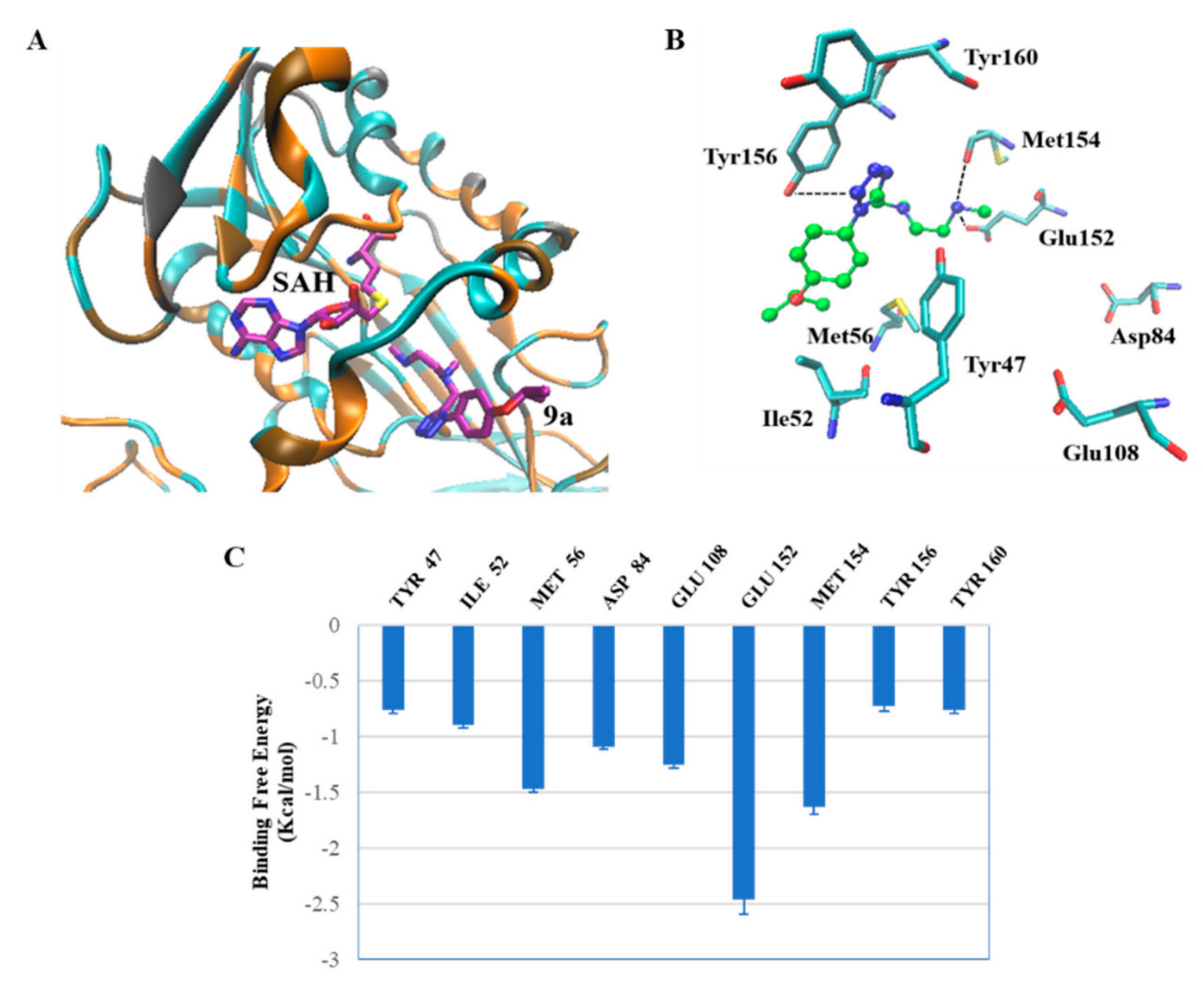
(**A**) The binding of SAH and compound **9a** (carbon atoms were colored in purple) with hPRMT1(gray = basic regions, ochre = acidic regions, orange = polar regions, cyan = nonpolar regions); (**B**) The interactions between key residues (carbon atoms were colored in cyan) and compound **9a** (carbon atoms were colored in green). The hydrogen bonds were represented by black dotted lines; (**C**) The residues whose binding free energy contributions were greater than −0.7 Kcal/mol.

To gain more detailed information regarding the interactions between the compound **9a** and hPRMT1-SAH complex, it was docked into the protein by using the position of substrate arginine binding site as the reference. There was 100 ns molecular dynamic simulation performed on the complex, and then the stable trajectory was collected for the binding free energy calculations (−9.64 kcal/mol) by using MM-PBSA and Normal Mode as shown in Table 2, which indicated a strong binding of compound **9a**. It was noticed that, in the stable MD trajectory, the compound **9a** occupied the substrate arginine binding site (Figure 6A), in which *N*, *N*’-dimethylethylenediamine mimicked the guanidyl group of the arginine substrate and bound closely to the SAH, and the binding pose of compound **9a** was stably maintained during the whole simulation process. The free energy decomposition was performed and the residues whose binding free energy contributions greater than –0.70 kcal/mol were recorded (Figure 6C). It was noticed that the Tyr47, Ile52, Met56, Asp84, Glu108, Glu152, Met154, Tyr156 and Tyr160 formed strong interactions with the compound **9a** (Figure 6B). Among these residues, Tyr47, Ile52, Met56 and Tyr156 formed strong van der Walls interactions, in which Tyr47 and Tyr156 formed π-π interactions with the aromatic ring of compound **9a**, and three negatively charged residues (Asp84, Glu108 and Glu152) contributed large electrostatic interactions because of the positive charges of compound **9a**. The MD simulations also showed that the compound **9a** could form three stable hydrogen bonds with Tyr156, Met154 and Glu152 along the stable trajectory. By using the molecular dynamic simulations, we believed that the binding site of compound **9a** is at the substrate arginine binding position, so that the methylation process could be disturbed. However, the molecular dynamic simulation results need to be further validated by experimental data, such as the key residues mutations in the active site or co-crystallization, which will be considered in our following work.

### 2.6. The Alteration of **9a** on PRMT1 Patterns at the Cellular Level

**Figure 7:**
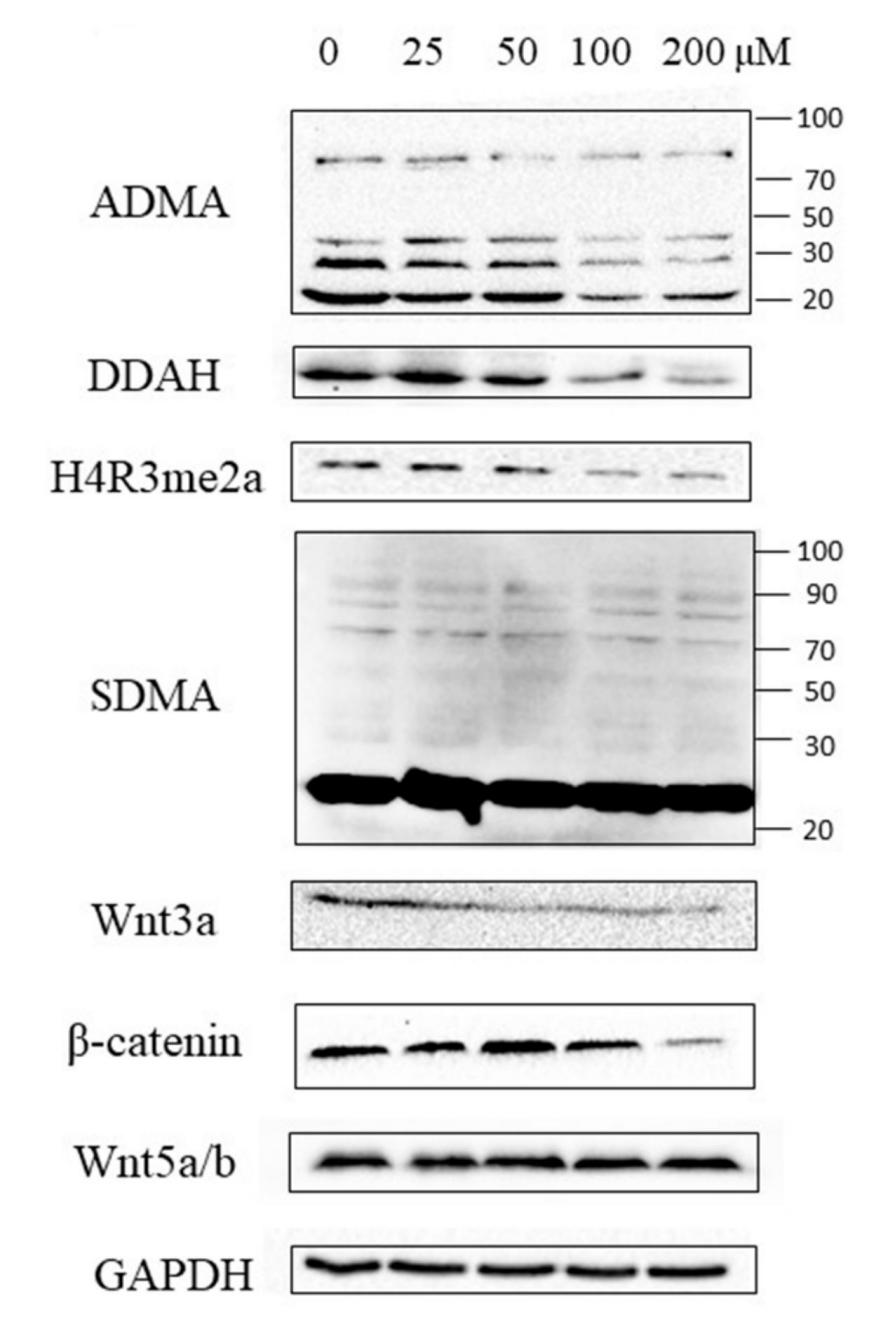
After the treatment of MDA-MB-231 cells with **9a** for 48 h, the western blotting results showed that the global levels of asymmetrical dimethylarginine (ADMA), dimethylaminohydrolases (DDAH) and H4R3me2a were significantly decreased in concentration-dependent manners, and the global level of symmetrical dimethylarginine (SDMA) was not significantly changed. On the Wnt pathway, the western blotting results showed that the global levels of Wnt3A and β-catenin were significantly decreased in concentration-dependent manners, while the level of Wnt5a/b was not changed. The experiments were repeated in triplicates.

Western blotting was performed to confirm that compound **9a** altered the arginine methylation patterns of PRMT1 in the highly metastatic breast cancer cell line MDA-MB-231, as it has been reported that the expression of PRMT1 is high in the MDA-MB-231 [29]. Before the western blotting experiment was performed, the potential cytotoxic effect of compound **9a** against MDA-MB-231 (breast cancer) cells was examined using WST-8/CCK8 cell viability assays. The results showed the IC_50_ of compound **9a** against MDA-MB-231 for 48 h was much higher than 500 μM. We then extended the time to 72 h, and the IC_50_ was obtained as 541.1 ± 6.5 μM. Therefore, the top concentration of compound **9a** for the western blotting experiment was chosen as 200 μM, and during the experiment, no obvious cell damage was observed. As shown in Figure 7, after the treatment of MDA-MB-231 cells with **9a** for 48 h, the global level of ADMA, which is mainly produced by the type I PRMTs, was significantly decreased in a concentration-dependent manner. It was also noticed that the level of dimethylarginine dimethylaminohydrolases (DDAH), which can metabolize more than 90% of ADMA [30], was significantly decreased in a concentration-dependent manner. The results showed that the decreasing of ADMA was because of the inhibition of PRMT1 rather than being metabolized by DDAH, which indicated that the compound **9a** influenced the PRMT-AMDA pathway. Asymmetric dimethylation of histone H4 at arginine 3 (H4R3me2as) is mediated by PRMT1, and it was observed that the compound **9a** decreased the global level of H4R3me2as in a concentration-dependent manner. The global level of SDMA, which was catalyzed by Type II PRMTs, was not significantly affected. By combining the above information, it could be confirmed that **9a** has significant influences on PRMT pathway at cellular levels.

It was reported that the PRMT1 is required for canonical Wnt signaling [31], and another recent paper reported that the depletion of PRMT1 blocked Wnt-induced micropinocytosis [32], which reminded us to see if the inhibition of PRMT1 by compound **9a** shows influences on the Wnt signaling pathways. The global levels of Wnt3a/β-catenin (Canonical Wnt signaling) and Wnt5a (Non-canonical Wnt signaling) were evaluated, in which the levels of Wnt3a and β-catenin were decreased in concentration-dependent manners, and the level of Wnt5a did not change significantly. The results suggested that the inhibition of PRMT1 by compound **9a** selectively down regulated the canonical Wnt signaling pathway, which was of potential medical interests. To further confirm the relationships between PRMT1 inhibitors and Wnt pathway, more factors or biomarkers need to be evaluated in the future work, such as matrix metallopeptidase 2 (MMP2), matrix metallopeptidase 9 (MMP9), and methylation of Ras GTPase-activating protein-binding protein 1 (G3BP1) etc.

## 3. Materials and Methods

### 3.1. General Information

All the required chemicals were purchased from commercial sources and used without further purification. TLC was performed on silica gel 60 pre-coated aluminium plates (0.20 mm thickness) from Macherey-Nagel and visualisation was accomplished with UV light (254 nm). Compounds were purified by flash column chromatography using 80–100 mesh silica gel. ^1^HNMR and ^13^CNMRspectra were obtained from Bruker AVANCE III 400 spectrometers using chloroform-d, DMSO-d6 or deuterium oxide as a solvent. The chemical shifts, given as δ values, were quoted in parts per million (ppm); ^1^HNMR chemical shifts were measured relative to internal tetramethylsilane; multiplicities quoted as singlet (s), doublet (d), triplet (t), quartet (q) or combinations thereof as appropriate. HRMS spectra were obtained on a Thermo Q-Exactive Orbitrap mass spectrometer. Melting points were determined on a WRS-1B apparatus without corrected.

### 3.2. Chemical Synthesis

#### 3.2.1. Synthesis of **9a**–**f** and **10a**–**e**

General procedure for the preparation of compounds **2a**–**f**.

Triethylamine (2.5 eq) was added to the substituted aniline **1a**–**f** (1.0 eq) dissolved in anhydrous dichloromethane (1.5 mL/mmol). The reaction mixture was cooled to 0 °C and ethyl oxalyl monochloride (1.2 eq) was added dropwise to the solution. Subsequently, the reaction was warmed to room temperature and stirred for 1 h. The reaction mixture was quenched with water and extracted with dichloromethane, the organic layer was dried over anhydrous Na_2_SO_4_, filtered and concentrated. The residue was purified by column chromatography on silica gel using EtOAc/petroleum as eluent to give **2a**–**f**.

Ethyl *N*-(4-isopropoxyphenyl)oxamate (**2a**)

Yellow solid. Yield: 94%. MP: 128.7–129.9 °C. ^1^H NMR (400 MHz, DMSO-*d*_6_) δ 10.67 (s, 1H, *-NH-*), 7.69–7.55 (m, 2H, *-ArH*), 6.98–6.77 (m, 2H, *-ArH*), 4.57 (Hept, J = 6.0 Hz, 1H, *-OCH(CH_3_)_2_*), 4.30 (q, *J* = 7.1 Hz, 2H, *-CH2-*), 1.31 (t, *J* = 7.1 Hz, 3H, *-CH3*), 1.26 (s, 3H, *-CH3*), 1.24 (s, 3H, *-CH3*).

Ethyl *N*-(3-isopropoxyphenyl)oxamate (**2b**)

Yellow oil. Yield: 92%. ^1^H NMR (400 MHz, Chloroform-*d*) δ 8.84 (s, 1H, *-NH-*), 7.39 (t, *J* = 2.2 Hz, 1H, *-ArH*), 7.26 (t, *J* = 8.1 Hz, 1H, *-ArH*), 7.09 (ddd, J = 8.1, 2.1, 1.0 Hz, 1H, *-ArH*), 6.74 (ddd, *J* = 8.3, 2.5, 0.9 Hz, 1H, *-ArH*), 4.58 (p, *J* = 6.1 Hz, 1H, *-OCH(CH_3_)_2_*), 4.44 (q, *J* = 7.1 Hz, 2H, *-CH_2_-*), 1.46 (t, *J* = 7.2 Hz, 3H, *-CH_3_*), 1.36 (d, *J* = 6.1 Hz, 6H, *-CH(CH_3_)_2_*).

Ethyl *N*-(4-methoxyphenyl)oxamate (**2c**)

Yellow solid. Yield: 94%. MP: 105.7–106.3 °C. ^1^H NMR (400 MHz, Chloroform-*d*) δ 8.81 (s, 1H, *-NH-*), 7.61–7.56 (m, 2H, *-ArH*), 6.95–6.90 (m, 2H, *-ArH*), 4.44 (q, *J* = 7.1 Hz, 2H, *-CH_2_-*), 3.83 (s, 3H, *-OCH_3_*), 1.45 (t, *J* = 7.1 Hz, 3H, *-CH_3_*).

Ethyl *N*-(3-methoxyphenyl)oxamate (**2d**)

Yellow solid. Yield: 95%. MP: 88.9–99.3 °C. ^1^H NMR (400 MHz, Chloroform-*d*) δ 8.87 (s, 1H, *-NH-*), 7.41 (t, *J* = 2.3 Hz, 1H, *-ArH*), 7.30 (d, *J* = 8.1 Hz, 1H, *-ArH*), 7.13 (ddd, *J* = 8.0, 2.0, 0.9 Hz, 1H, *-ArH*), 6.77 (ddd, *J* = 8.3, 2.5, 0.9 Hz, 1H, *-ArH*), 4.44 (q, *J* = 7.2 Hz, 2H, *-CH_2_-*), 3.84 (s, 3H, *-OCH_3_*), 1.46 (t, *J* = 7.2 Hz, 3H, *-OCH_3_*), 1.46 (t, *J* = 7.2 Hz, 3H, *-CH_3_*).

Ethyl *N*-(3-trifluoromethoxyphenyl)oxamate (**2e**)

Yellow oil. Yield: 90%. ^1^H NMR (400 MHz, Chloroform-*d*) δ 8.96 (s, 1H, *-NH-*), 7.70 (d, *J* = 2.5 Hz, 1H, *-ArH*), 7.54 (ddd, *J* = 8.3, 2.1, 0.9 Hz, 1H, *-ArH*), 7.42 (t, *J* = 8.2 Hz, 1H, *-ArH*), 7.08 (ddt, *J* = 8.3, 2.3, 1.1 Hz, 1H, *-ArH*), 4.46 (q, *J* = 7.2 Hz, 2H, *-CH2-*), 1.46 (t, *J* = 7.2 Hz, 3H, *-CH3*).

Ethyl *N*-(4-trifluoromethoxyphenyl)oxamate (**2f**)

Yellow solid. Yield: 92%. MP: 131.2–131.9 °C. ^1^H NMR (400 MHz, DMSO-*d*_6_) δ 11.01 (s, 1H, *-NH-*), 7.90–7.85 (m, 2H, *-ArH*), 7.42–7.36 (m, 2H, *-ArH*), 4.32 (q, *J* = 7.1 Hz, 2H, *-CH2-*), 1.32 (t, *J* = 7.1 Hz, 3H, *-CH3*).

General procedure for the preparation of compounds **3a**–**f**.

**2a**–**f** (1.0 eq) was dissolved in CCl_4_ (2.0 mL/mmol), and a solution of triphenylphosphine in CCl_4_ (0.8 mL/mmol) was added dropwise to the reaction flask at the room temperature. The reaction was refluxed and stirred for 6 h, and then cooled to the room temperature. The precipitate was filtered off and washed with CCl_4_. The filtrate was concentrated in vacuo without further purification to give **3a**–**f**.

General procedure for the preparation of compounds **4a**–**f**.

To a solution of **3a**–**f** (1.0 eq) in acetonitrile (1 mL/mmol) was added sodium azide (1.5 eq), and the reaction was stirred at the room temperature for 3 h and monitored by TLC. The mixture was quenched with ice water, concentrated and extracted with ethyl acetate. The combined organic layers were washed with water, and dried over anhydrous Na_2_SO4, filtered and concentrated. The residue was purified by column chromatography on silica gel using EtOAc/petroleum as eluent to give **4a**–**f**.

Ethyl 1-(4-isopropoxyphenyl)-1H-tetrazole-5-carboxylate (**4a**)

White solid. Yield: 65%. MP: 150.2–151.9 °C. ^1^H NMR (400 MHz, Chloroform-*d*) δ 7.44–7.35 (m, 2H, *-ArH*), 7.06–6.99 (m, 2H, *-ArH*), 4.64 (p, *J* = 6.1 Hz, 1H, *-OCH(CH_3_)_2_*), 4.46 (q, *J* = 7.1 Hz, 2H, *-CH2-*), 1.39 (dd, *J* = 7.9, 6.6 Hz, 9H, *-CH_3_*).

Ethyl 1-(3-isopropoxyphenyl)-1H-tetrazole-5-carboxylate (**4b**)

Yellow oil. Yield: 63%. ^1^H NMR (400 MHz, DMSO-*d*_6_) δ 7.48 (t, *J* = 8.1 Hz, 1H, *-ArH*), 7.35 (t, *J* = 2.2 Hz, 1H, *-ArH*), 7.19 (dddd, *J* = 11.7, 8.4, 2.2, 0.9 Hz, 2H, *-ArH*), 4.65 (h, *J* = 6.0 Hz, 1H, *-OCH(CH_3_)_2_*), 4.33 (t, *J* = 7.1 Hz, 2H, *-CH_2_-*), 1.30 (s, 3H, *-CH_3_*), 1.28 (s, 3H, *-CH_3_*), 1.20 (t, *J* = 7.1 Hz, 3H, *-CH_3_*).

Ethyl 1-(4-methoxyphenyl)-1H-tetrazole-5-carboxylate (**4c**)

White solid. Yield: 65%. MP: 75.9–76.3 °C. ^1^H NMR (400 MHz, Chloroform-*d*) δ 7.46–7.41 (m, 2H, *-ArH*), 7.10–7.05 (m, 2H, *-ArH*), 4.47 (q, *J* = 7.1 Hz, 2H, *-CH_2_-*), 3.92 (s, 3H, *-OCH_3_*), 1.41 (t, *J* = 7.1 Hz, 3H, *-CH_3_*).

Ethyl 1-(3-methoxyphenyl)-1H-tetrazole-5-carboxylate (**4d**)

White solid. Yield: 64%. MP: 70.1–72.0 °C. ^1^H NMR (400 MHz, Chloroform-*d*) δ 7.49 (t, *J* = 8.1 Hz, 1H, *-ArH*), 7.16 (ddd, *J* = 8.5, 2.5, 0.9 Hz, 1H, *-ArH*), 7.08 (ddd, *J* = 7.8, 2.0, 0.9 Hz, 1H, *-ArH*), 7.05 (t, *J* = 2.2 Hz, 1H, *-ArH*), 4.48 (q, *J* = 7.1 Hz, 2H, *-CH_2_-*), 3.89 (s, 3H, *-OCH_3_*), 1.40 (t, *J* = 7.1 Hz, 3H, *-CH_3_*).

Ethyl 1-(3-trifluoromethoxyphenyl)-1H-tetrazole-5-carboxylate (**4e**)

Yellow solid. Yield: 64%. MP: 125.2–126.0 °C. ^1^H NMR (400 MHz, Chloroform-*d*) δ 7.66 (dd, *J* = 8.7, 7.7 Hz, 1H, *-ArH*), 7.51 (dd, *J* = 7.9, 2.2 Hz, 2H, *-ArH*), 7.46 (q, *J* = 1.9 Hz, 1H, *-ArH*), 4.49 (q, *J* = 7.1 Hz, 2H, *-CH2-*), 1.41 (t, *J* = 7.1 Hz, 3H, *-CH_3_*).

Ethyl 1-(4-trifluoromethoxyphenyl)-1H-tetrazole-5-carboxylate (**4f**)

Yellow solid. Yield: 65%. MP: 68.2–69.0 °C. ^1^H NMR (400 MHz, DMSO-*d*_6_) δ 7.96–7.84 (m, 2H, *-ArH*), 7.71–7.60 (m, 2H, *-ArH*), 4.32 (q, *J* = 7.1 Hz, 2H, *-CH_2_-*), 1.19 (t, *J* = 7.1 Hz, 3H, *-CH_3_*).

General procedure for the preparation of compounds **5a**–**f**.

To a solution of **4a**–**f** (1.0 eq) in anhydrous dichloromethane (1.8 mL/mmol) was added diisobutyl aluminium hydride (1.0M in hexanes, 2.0 eq) dropwise at −78 °C and the reaction was stirred for 30 min. The mixture was quenched with methanol, concentrated and extracted with ethyl acetate. The combined organic layers were washed with water, 1 M HCl and dried over anhydrous Na_2_SO4, filtered and concentrated. The residue was purified by column chromatography on silica gel using EtOAc/petroleum as eluent to give **5a**–**f.**

1-(4-Isopropoxyphenyl)-1H-tetrazole-5-carbaldehyde (**5a**)

White solid. Yield: 80%. MP: 122.5–123.1 °C. ^1^H NMR (400 MHz, DMSO-*d*_6_) δ 10.05 (s, 1H, *-CHO*), 7.67–7.63 (m, 2H, *-ArH*), 7.17–7.13 (m, 2H, *-ArH*), 4.79–4.72 (m, 1H, *-OCH(CH_3_)_2_*), 1.33 (s, 3H, *-CH_3_*), 1.32 (s, 3H, *-CH_3_*).

1-(3-Isopropoxyphenyl)-1H-tetrazole-5-carbaldehyde (**5b**)

Colorless oil. Yield: 75%. ^1^H NMR (400 MHz, DMSO-*d*_6_) δ 10.07 (s, 1H, *-CHO*), 7.53 (t, *J* = 8.1 Hz, 1H, *-ArH*), 7.38 (t, *J* = 2.2 Hz, 1H, *-ArH*), 7.29–7.25 (m, 2H, *-ArH*), 4.69–4.64 (m, 1H, *-OCH(CH_3_)_2_*), 1.31 (d, *J* = 0.8 Hz, 3H, *-CH_3_*), 1.29 (d, *J* = 0.8 Hz, 3H, *-CH_3_*).

1-(4-Methoxyphenyl)-1H-tetrazole-5-carbaldehyde (**5c**)

Colorless oil. Yield: 77%. ^1^H NMR (400 MHz, Chloroform-*d*) δ 10.33 (s, 1H, *-CHO*), 7.51–7.47 (m, 2H, *-ArH*), 7.11–7.07 (m, 2H, *-ArH*), 3.93 (s, 3H, *-OCH_3_*).

1-(3-Methoxyphenyl)-1H-tetrazole-5-carbaldehyde (**5d**)

Colorless oil. Yield: 75%. ^1^H NMR (400 MHz, DMSO-*d*_6_) δ 10.07 (s, 1H, *-CHO*) 7.59–7.54 (t, 1H, *-ArH*), 7.41 (t, *J* = 2.2 Hz, 1H, *-ArH*), 7.31 (dddd, *J* = 7.9, 6.8, 2.0, 0.9 Hz, 2H, *-ArH*), 3.83 (s, 3H, *-OCH_3_*).

1-(3-Trifluoromethoxyphenyl)-1H-tetrazole-5-carbaldehyde (**5e**)

Yellow solid. Yield: 78%. MP: 97.6–98.0 °C. ^1^H NMR (400 MHz, DMSO-*d*_6_) δ 10.11 (s, 1H, *-CHO*), 7.96–7.91 (m, 2H, *-ArH*), 7.75–7.70 (m, 1H, *-ArH*), 7.65 (ddt, *J* = 8.0, 2.3, 1.1 Hz, 1H, *-ArH*).

1-(4-Trifluoromethoxyphenyl)-1H-tetrazole-5-carbaldehyde (**5f**)

Yellow solid. Yield: 80%. MP: 118.7–119.5 °C. ^1^H NMR (400 MHz, DMSO-*d*_6_) δ 10.10 (s, 1H, *-CHO*), 7.94–7.91 (m, 2H, *-ArH*), 7.70 (dtd, *J* = 8.1, 2.2, 1.2 Hz, 2H, *-ArH*).

General procedure for the preparation of compounds **7a**–**f** and **8a**–**e**.

To a solution of **5a**–**f** (1.0 eq) in 1,2-Dichloroethane (8.5 mL/mmol) was added sodium triacetoxyborohydride (2.0 eq) and **6a** or **6b** (1.0 eq) and the reaction was stirred for 12 h at room temperature. The reaction mixture was diluted with water and extracted with dichloromethane. The dichloromethane layer was dried over anhydrous Na_2_SO4, filtered and concentrated. The residue was purified by column chromatography on silica gel using EtOAc/petroleum as eluent to give **7a–f** or **8a**–**e**.

*N*-tert-Butyloxycarbonyl-*N*-methyl-*N*’-(1-(4-isopropoxyphenyl)-1H-tetrazol-5-yl-methyl)-*N*’-methyl-ethylenediamine (**7a**)

Colorless oil. Yield: 85%. ^1^H NMR (400 MHz, Chloroform-*d*) δ 7.59 (d, *J* = 8.5 Hz, 2H, *-ArH*), 7.05 (d, *J* = 8.9 Hz, 2H, *-ArH*), 4.65 (p, *J* = 6.0 Hz, 1H, *-OCH(CH_3_)_2_*), 3.82 (s, 2H, *-CH_2_-*), 3.31 (s, 2H, *-CH_2_-*), 2.78 (s, 3H, *-CH_3_*), 2.38 (s, 3H, *-CH_3_*), 1.44 (s, 9H, *-C(CH_3_)_3_*), 1.41 (s, 3H, *-CH_3_*), 1.40 (s, 3H, *-CH_3_*).

*N*-tert-Butyloxycarbonyl-*N*-methyl-*N*-(1-(3-isopropoxyphenyl)-1H-tetrazol-5-yl-methyl)-*N*’-methyl-ethylenediamine (**7b**)

Colorless oil. Yield: 82%. ^1^H NMR (400 MHz, DMSO-*d*_6_) δ 7.52 (t, *J* = 8.1 Hz, 1H, *-ArH*), 7.35–7.24 (m, 2H, *-ArH*), 7.17 (ddd, *J* = 8.4, 2.5, 0.9 Hz, 1H, *-ArH*), 4.71 (p, *J* = 6.0 Hz, 1H, *-OCH(CH_3_)_2_*), 3.11 (t, *J* = 6.4 Hz, 2H, *-CH_2_-*), 2.63 (d, *J* = 6.4 Hz, 3H, *-CH_3_*), 2.48 (t, *J* = 6.6 Hz, 2H, *-CH_2_-*), 2.20 (d, *J* = 6.4 Hz, 3H, *-CH_3_*), 1.31 (dd, *J* = 12.4, 8.1 Hz, 15H, *-CH_3_, -C(CH_3_)_3_*).

*N*-tert-Butyloxycarbonyl-*N*-methyl-*N*’-(1-(4-methoxyphenyl)-1H-tetrazol-5-ylmethyl)-*N*’-methyl-ethylenediamine (**7c**)

Colorless oil. Yield: 85%. ^1^H NMR (400 MHz, DMSO-*d*_6_) δ 7.66 (dd, *J* = 8.3, 2.8 Hz, 2H, *-ArH*), 7.21–7.11 (m, 2H, *-ArH*), 3.85 (s, 3H, *-CH_3_*), 3.83 (s, 2H, *-CH_2_-*), 3.15 (d, *J* = 6.3 Hz, 2H, *-CH_2_-*), 2.64 (d, *J* = 9.5 Hz, 3H, *-CH_3_*), 2.47 (d, *J* = 6.5 Hz, 2H, *-CH_2_-*), 2.18 (d, *J* = 6.8 Hz, 3H, *-CH_3_*), 1.33 (d, *J* = 12.1 Hz, 9H, *-C(CH_3_)_3_*).

*N*-tert-Butyloxycarbonyl-*N*-methyl-*N*’-(1-(3-methoxyphenyl)-1H-tetrazol-5-yl-methyl)-*N*’-methyl-ethylenediamine (**7d**)

Colorless oil. Yield: 83%. ^1^H NMR (400 MHz, DMSO-*d*_6_) δ 7.55 (t, *J* = 8.1 Hz, 1H, *-ArH*), 7.39–7.30 (m, 2H, *-ArH*), 7.20 (ddd, *J* = 8.4, 2.6, 0.9 Hz, 1H, *-ArH*), 3.90 (s, 2H, *-CH_2_-*), 3.84 (s, 3H, *-CH_3_*), 3.13 (d, *J* = 7.2 Hz, 2H, *-CH_2_-*), 2.62 (d, *J* = 6.6 Hz, 3H, *-CH_3_*), 2.48 (d, *J* = 6.6 Hz, 2H, *-CH_2_-*), 2.21 (s, 3H, *-CH_3_*), 1.32 (d, *J* = 10.5 Hz, 9H, *-C(CH_3_)_3_*).

*N*-tert-Butyloxycarbonyl-*N*-methyl-*N*’-(1-(3-trifluoromethoxyphenyl)-1H-tetrazol-5-yl-methyl)-*N*’-methyl-ethylenediamine (**7e**)

Colorless oil. Yield: 82%. ^1^H NMR (400 MHz, DMSO-*d*_6_) δ 7.93 (s, 1H, *-ArH*), 7.87–7.82 (m, 1H, *-ArH*), 7.79 (t, *J* = 8.1 Hz, 1H, *-ArH*), 7.68 (ddt, *J* = 8.0, 2.4, 1.2 Hz, 1H, *-ArH*), 3.94 (s, 2H, *-CH_2_-*), 3.09 (t, *J* = 6.3 Hz, 2H, *-CH_2_-*), 2.59 (d, *J* = 5.4 Hz, 3H, *-CH_3_*), 2.46 (t, *J* = 6.6 Hz, 2H, *-CH_2_-*), 2.19 (s, 3H, *-CH_3_*), 1.31 (d, *J* = 9.5 Hz, 9H, *-C(CH_3_)_3_*).

*N*-tert-Butyloxycarbonyl-*N*-methyl-*N*’-(1-(4-trifluoromethoxyphenyl)-1H-tetrazol-5-yl-methyl)-*N*’-methyl-ethylenediamine (**7f**)

Colorless oil. Yield: 84%. ^1^H NMR (400 MHz, DMSO-*d*_6_) δ 7.94 (d, *J* = 8.3 Hz, 2H, *-ArH*), 7.69–7.64 (m, 2H, *-ArH*), 3.91 (s, 2H, *-CH_2_-*), 3.13 (t, *J* = 6.4 Hz, 2H, *-CH_2_-*), 2.61 (d, *J* = 6.3 Hz, 3H, *-CH_3_*), 2.48 (d, *J* = 6.5 Hz, 2H, *-CH_2_-*), 2.18 (d, *J* = 7.0 Hz, 3H, *-CH_3_*), 1.32 (d, *J* = 11.3 Hz, 9H, *-C(CH_3_)_3_*).

*N*-tert-Butyloxycarbonyl-*N*-methyl-*N*’-(1-(4-isopropoxyphenyl)-1H-tetrazol-5-yl-methyl)-*N*’-methyl-1,3-propylenediamine (**8a**)

Colorless oil. Yield: 80%. ^1^H NMR (400 MHz, DMSO-*d*_6_) δ 7.61 (dd, *J* = 9.1, 2.5 Hz, 2H, *-ArH*), 7.15–7.10 (m, 2H, *-ArH*), 4.76–4.69 (m, 1H, *-OCH(CH_3_)_2_*), 3.81 (s, 2H, *-CH_2_-*), 2.92 (s, 2H, *-CH_2_-*), 2.66 (s, 3H, *-CH_3_*), 2.29 (t, *J* = 7.0 Hz, 2H, *-CH_2_-*), 2.10 (s, 3H, *-CH_3_*), 1.45 (t, *J* = 7.2 Hz, 2H, *-CH_2_-*), 1.34 (d, *J* = 11.2 Hz, 9H, *-C(CH_3_)_3_*), 1.31 (d, *J* = 1.5 Hz, 3H, *-CH_3_*), 1.30 (s, 3H, *-CH_3_*).

*N*-tert-Butyloxycarbonyl-*N*-methyl-*N*’-(1-(3-isopropoxyphenyl)-1H-tetrazol-5-yl-methyl)-*N*’-methyl-1,3-propylenediamine (**8b**)

Colorless oil. Yield: 77%. ^1^H NMR (400 MHz, DMSO-*d*_6_) δ 7.51 (t, *J* = 8.2 Hz, 1H, *-ArH*), 7.33 (d, *J* = 9.6 Hz, 1H, *-ArH*), 7.28–7.22 (m, 1H, *-ArH*), 7.21–7.14 (m, 1H, *-ArH*), 4.70 (p, *J* = 6.0 Hz, 1H, *-OCH(CH_3_)_2_*), 3.86 (s, 2H, *-CH_2_-*), 2.94 (s, 2H, *-CH_2_-*), 2.67 (d, *J* = 4.2 Hz, 3H, *-CH_3_*), 2.29 (t, *J* = 7.0 Hz, 2H, *-CH_2_-*), 2.11 (s, 3H, *-CH_3_*), 1.46 (t, *J* = 7.1 Hz, 2H, *-CH_2_-*), 1.33 (d, *J* = 15.1 Hz, 9H, *-C(CH_3_)_3_*), 1.30 (s, 3H, *-CH_3_*), 1.28 (s, 3H, *-CH_3_*).

*N*-tert-Butyloxycarbonyl-*N*-methyl-*N*’-(1-(4-methoxyphenyl)-1H-tetrazol-5-yl-methyl)-*N*’-methyl-1,3-propylenediamine (**8c**)

Colorless oil. Yield: 75%. ^1^H NMR (400 MHz, DMSO-*d*_6_) δ 7.67–7.62 (m, 2H, *-ArH*), 7.18–7.14 (m, 2H, *-ArH*), 3.85 (d, *J* = 4.2 Hz, 3H, *-CH_3_*), 3.81 (s, 2H, *-CH_2_-*), 2.94 (s, 2H, *-CH_2_-*), 2.67 (s, 3H, *-CH_3_*), 2.28 (t, *J* = 7.1 Hz, 2H, *-CH_2_-*), 2.10 (s, 3H, *-CH_3_*), 1.45 (s, 2H, *-CH_2_-*), 1.36 (s, 9H, *-C(CH_3_)_3_*).

*N*-tert-Butyloxycarbonyl-*N*-methyl-N’-(1-(3-methoxyphenyl)-1H-tetrazol-5-yl-methyl)-N’-methyl-1,3-propylenediamine (**8d**)

Colorless oil. Yield: 75%. ^1^H NMR (400 MHz, DMSO-*d*_6_) δ 7.54 (t, *J* = 8.2 Hz, 1H, *-ArH*), 7.39 (t, *J* = 2.3 Hz, 1H, *-ArH*), 7.30 (dddd, *J* = 6.8, 2.8, 1.9, 0.9 Hz, 1H, *-ArH*), 7.23–7.17 (m, 1H, *-ArH*), 3.87 (s, 2H, *-CH_2_-*), 3.83 (s, 3H, *-CH_3_*), 2.93 (s, 2H, *-CH_2_-*), 2.66 (s, 3H, *-CH_3_*), 2.30 (t, *J* = 7.1 Hz, 2H, *-CH_2_-*), 2.12 (s, 3H, *-CH_3_*), 1.46 (t, *J* = 7.2 Hz, 2H, *-CH_2_-*), 1.35 (s, 9H, *-C(CH_3_)_3_*).

*N*-tert-Butyloxycarbonyl-*N*-methyl-*N*’-(1-(3-trifluoromethoxyphenyl)-1H-tetrazol-5-yl-methyl)-*N*’-methyl-1,3-propylenediamine (**8e**)

Colorless oil. Yield: 75%. ^1^H NMR (400 MHz, DMSO-*d*_6_) δ 7.98 (s, 1H, *-ArH*), 7.83 (ddd, *J* = 8.0, 4.1, 2.0 Hz, 2H, *-ArH*), 7.68 (dtq, *J* = 7.3, 2.5, 1.2 Hz, 1H, *-ArH*), 3.90 (s, 2H, *-CH_2_-*), 2.87 (s, 2H, *-CH_2_-*), 2.65 (s, 3H, *-CH_3_*), 2.27 (t, *J* = 7.1 Hz, 2H, *-CH_2_-*), 2.08 (s, 3H, *-CH_3_*), 1.42 (d, *J* = 7.4 Hz, 2H, *-CH_2_-*), 1.35 (s, 9H, *-C(CH_3_)_3_*).

General procedure for the preparation of compounds **9a**–**f** and **10a**–**e**.

Compounds **7a**–**f** or **8a**–**e** (0.51 mmol) was added to saturated hydrochloric acid ethanol solution (10 mL) at room temperature and stirred for 30 min. The reaction solution was filtered and washed with diethylether (20 mL) to give **9a**–**f** or **10a**–**e**.

*N*,*N*’-Dimethyl-*N*’-(1-(4-isopropoxyphenyl)-1H-tetrazol-5-yl-methyl)ethylenediamine hydrochloride (**9a**)

White solid. Yield: 98%. MP: 187.4–189.4 °C. ^1^H NMR (400 MHz, Deuterium Oxide) δ 7.49–7.44 (m, 2H, *-ArH*), 7.19–7.13 (m, 2H, *-ArH*), 4.74 (p, *J* = 5.5, 4.9 Hz, 1H, *-OCH(CH_3_)_2_*), 4.35 (s, 2H, *-CH_2_-*), 3.21 (dd, *J* = 14.9, 6.8 Hz, 2H, *-CH_2_-*), 3.15–3.01 (m, 2H, *-CH_2_-*), 2.64 (dd, *J* = 4.9, 2.1 Hz, 3H, *-CH_3_*), 2.48 (d, *J* = 37.8 Hz, 3H, *-CH_3_*), 1.31 (s, 3H, *-CH_3_*), 1.30 (s, 3H, *-CH_3_*). ^13^C NMR (101 MHz, Deuterium Oxide) δ 159.2, 150.1, 126.7, 124.9, 117.1, 71.9, 51.8, 49.1, 44.2, 41.3, 33.0, 20.9. HRMS (ESI) m/z [M+H]^+^ Calcd for C_15_H_25_N_6_O^+^: 305.20844. Found: 305.20831.

*N*,N’-Dimethyl-*N*’-(1-(3-isopropoxyphenyl)-1H-tetrazol-5-yl-methyl)ethylenediamine hydrochloride (**9b**)

Colorless oil. Yield: 95%. ^1^H NMR (400 MHz, Deuterium Oxide) δ 7.49 (ddd, *J* = 8.4, 7.4, 0.9 Hz, 1H, *-ArH*), 7.18 (ddd, *J* = 8.5, 2.3, 1.1 Hz, 1H, *-ArH*), 7.09–7.04 (m, 2H, *-ArH*), 4.63 (p, *J* = 6.1 Hz, 1H, *-OCH(CH_3_)_2_*), 4.37 (s, 2H, *-CH_2_-*), 3.22 (td, *J* = 6.2, 1.3 Hz, 2H, *-CH_2_-*), 3.13 (td, *J* = 6.4, 1.3 Hz, 2H, *-CH_2_-*), 2.61 (s, 3H, *-CH_3_*), 2.52 (s, 3H, *-CH_3_*), 1.24 (s, 3H, *-CH_3_*), 1.22 (s, 3H, *-CH_3_*). ^13^C NMR (101 MHz, Deuterium Oxide) δ 158.1, 133.1, 131.3, 119.1, 117.3, 112.8, 72.1, 51.8, 49.2, 44.2, 41.3, 33.0, 20.9. HRMS (ESI) m/z [M+H]^+^ Calcd for C_15_H_25_N_6_O^+^: 305.20844. Found: 305.20828.

*N*,*N*’-Dimethyl-*N*’-(1-(4-methoxyphenyl)-1H-tetrazol-5-yl-methyl)ethylenediamine hydrochloride (**9c**)

White solid. Yield: 95%. MP: 163.2–163.6 °C. ^1^H NMR (400 MHz, Deuterium Oxide) δ 7.45–7.40 (m, 2H, *-ArH*), 7.14–7.10 (m, 2H, *-ArH*), 4.51 (s, 2H, *-CH_2_-*), 3.81 (s, 3H, *-CH_3_*), 3.31 (s, 4H, *-CH_2_-*), 2.67 (s, 3H, *-CH_3_*), 2.64 (d, *J* = 0.7 Hz, 3H, *-CH_3_*). ^13^C NMR (101 MHz, Deuterium Oxide) δ 161.1, 149.8, 126.6, 124.9, 115.3, 55.7, 51.8, 49.1, 44.00, 41.4, 33.0. HRMS (ESI) m/z [M+H]^+^ Calcd for C_13_H_21_N_6_O^+^: 277.17714. Found: 277.17715.

*N*,*N*’-Dimethyl-*N*’-(1-(3-methoxyphenyl)-1H-tetrazol-5-yl-methyl)ethylenediamine hydrochloride (**9d**)

White solid. Yield: 98%. MP: 185.6–186.4 °C. ^1^H NMR (400 MHz, Deuterium Oxide) δ 7.52–7.47 (m, 1H, *-ArH*), 7.18 (ddd, *J* = 8.5, 2.5, 0.9 Hz, 1H, *-ArH*), 7.10–7.05 (m, 2H, *-ArH*), 4.47 (s, 2H, *-CH_2_-*), 3.77 (s, 3H, *-CH_3_*), 3.29–3.20 (m, 4H, *-CH_2_-*), 2.62 (s, 3H, *-CH_3_*), 2.60 (s, 3H, *-CH_3_*). ^13^C NMR (101 MHz, Deuterium Oxide) δ 160.1, 149.7, 133.0, 131.2, 117.2, 117.1, 110.9, 55.7, 51.9, 49.2, 44.1, 41.4, 33.0. HRMS (ESI) m/z [M+H]^+^ Calcd for C_13_H_21_N_6_O^+^: 277.17714. Found: 277.17703.

*N*,*N*’-Dimethyl-*N*’-(1-(3-trifluoromethoxyphenyl)-1H-tetrazol-5-yl-methyl)ethylenediamine hydrochloride (**9e**)

White solid. Yield: 97%. MP: 185.6–186.4 °C. ^1^H NMR (400 MHz, Deuterium Oxide) δ 7.66 (td, *J* = 8.1, 1.5 Hz, 1H, *-ArH*), 7.58–7.52 (m, 2H, *-ArH*), 7.52–7.48 (m, 1H, *-ArH*), 4.49 (s, 2H, *-CH_2_-*), 3.28 (d, *J* = 4.2 Hz, 2H, *-CH_2_-*), 3.26 (d, *J* = 3.9 Hz, 2H, *-CH_2_-*), 2.63 (s, 3H, *-CH_3_*), 2.62 (s, 3H, *-CH_3_*). ^13^C NMR (101 MHz, Deuterium Oxide) δ 150.7, 149.4, 133.2, 131.8, 123.9, 123.5, 118.1, 51.9, 49.4, 44.5, 41.3, 32.9. HRMS (ESI) m/z [M+H]^+^ Calcd for C_13_H_18_F_3_N_6_O^+^: 331.14887. Found: 331.14871.

*N*,*N*’-Dimethyl-*N*’-(1-(4-trifluoromethoxyphenyl)-1H-tetrazol-5-yl-methyl)ethylenediamine hydrochloride (**9f**)

White solid. Yield: 98%. MP: 194.3–195.2 °C. ^1^H NMR (400 MHz, Deuterium Oxide) δ 7.61–7.56 (m, 2H, *-ArH*), 7.51–7.46 (m, 2H, *-ArH*), 4.25 (s, 2H, *-CH_2_-*), 3.15 (t, *J* = 6.4 Hz, 2H, *-CH_2_-*), 3.02 (t, *J* = 6.4 Hz, 2H, *-CH_2_-*), 2.58 (s, 3H, *-CH_3_*), 2.42 (s, 3H, *-CH_3_*). ^13^C NMR (101 MHz, Deuterium Oxide) δ 150.6, 150.3, 130.6, 126.9, 122.5, 51.9, 49.3, 44.3, 41.3, 33.0. HRMS (ESI) m/z [M+H]^+^ Calcd for C_13_H_18_F_3_N_6_O^+^: 331.14887. Found: 331.14871.

*N*,*N*’-Dimethyl-*N*’-(1-(4-isopropoxyphenyl)-1H-tetrazol-5-yl-methyl)-1,3-propylenediamine hydrochloride (**10a**)

Colorless oil. Yield: 94%. ^1^H NMR (400 MHz, Deuterium Oxide) δ 7.43–7.39 (m, 2H, *-ArH*), 7.14–7.09 (m, 2H, *-ArH*), 4.76 (d, *J* = 1.2 Hz, 2H, *-CH_2_-*), 4.67 (d, *J* = 6.1 Hz, 1H, *-OCH(CH_3_)_2_*), 3.36–3.27 (m, 2H, *-CH_2_-*), 3.05–2.97 (m, 2H, *-CH_2_-*), 2.94–2.89 (m, 3H, *-CH_3_*), 2.61 (s, 3H, *-CH_3_*), 2.14–2.03 (m, 2H, *-CH_2_-*), 1.26 (s, 3H, *-CH_3_*), 1.25 (s, 3H, *-CH_3_*). ^13^C NMR (101 MHz, Deuterium Oxide) δ 159.4, 148.0, 126.7, 124.5, 117.2, 71.9, 53.5, 48.1, 45.4, 41.1, 32.71, 20.9. HRMS (ESI) m/z [M+H]^+^ Calcd for C_16_H_27_N_6_O^+^: 319.22409. Found: 319.22406.

*N*,*N*’-Dimethyl-*N*’-(1-(3-isopropoxyphenyl)-1H-tetrazol-5-yl-methyl)-1,3-propylenediamine hydrochloride (**10b**)

Colorless oil. Yield: 92%. ^1^H NMR (400 MHz, Deuterium Oxide) δ 7.50 (t, *J* = 8.1 Hz, 1H, *-ArH*), 7.20 (ddd, *J* = 8.5, 2.4, 0.9 Hz, 1H, *-ArH*), 7.09–7.03 (m, 2H, *-ArH*), 4.79 (s, 2H, *-CH_2_-*), 4.68–4.60 (m, 1H, *-OCH(CH_3_)_2_*), 3.36–3.28 (m, 2H, *-CH_2_-*), 3.04–2.96 (m, 2H, *-CH_2_-*), 2.91 (s, 3H, *-CH_3_*), 2.61 (s, 3H, *-CH_3_*), 2.15–2.04 (m, 2H, *-CH_2_-*), 1.24 (s, 3H, *-CH_3_*), 1.23 (s, 3H, *-CH_3_*). ^13^C NMR (101 MHz, Deuterium Oxide) δ 158.2, 147.7, 132.7, 131.4, 119.3, 117.3, 112.8, 72.1, 53.6, 48.2, 45.4, 41.1, 32.7, 20.9, 20.8. HRMS (ESI) m/z [M+H]^+^ Calcd for C_16_H_27_N_6_O^+^: 319.22409. Found: 319.22405.

*N*,*N*’-Dimethyl-*N*’-(1-(4-methoxyphenyl)-1H-tetrazol-5-yl-methyl)-1,3-propylenediamine hydrochloride (**10c**)

White solid. Yield: 94%. MP: 188.2–190.1 °C. ^1^H NMR (400 MHz, Deuterium Oxide) δ 7.48–7.42 (m, 2H, *-ArH*), 7.19–7.12 (m, 2H, *-ArH*), 4.73 (s, 2H, *-CH_2_-*), 3.84 (s, 3H, *-CH_3_*), 3.32–3.24 (m, 2H, *-CH_2_-*), 3.05–2.98 (m, 2H, *-CH_2_-*), 2.88 (s, 3H, *-CH_3_*), 2.64 (s, 3H, *-CH_3_*), 2.14–2.04 (m, 2H, *-CH_2_-*). ^13^C NMR (101 MHz, Deuterium Oxide) δ 148.1, 126.7, 124.5, 115.4, 55.7, 53.5, 48.1, 45.4, 41.1, 32.7, 20.9. HRMS (ESI) m/z [M+H]^+^ Calcd for C_14_H_23_N_6_O^+^: 291.19279. Found: 291.19271.

*N*,*N*’-Dimethyl-*N*’-(1-(3-methoxyphenyl)-1H-tetrazol-5-yl-methyl)-1,3-propylenediamine hydrochloride (**10d**)

Colorless oil. Yield: 92%. ^1^H NMR (400 MHz, Deuterium Oxide) δ 7.51 (dd, *J* = 8.5, 7.8 Hz, 1H, *-ArH*), 7.20 (ddd, *J* = 8.6, 2.5, 0.9 Hz, 1H, *-ArH*), 7.09 (t, *J* = 2.2 Hz, 1H, *-ArH*), 7.06 (ddd, *J* = 7.8, 2.1, 0.9 Hz, 1H, *-ArH*), 4.80 (d, *J* = 1.3 Hz, 2H, *-CH_2_-*), 3.78 (s, 3H, *-CH_3_*), 3.36–3.29 (m, 2H, *-CH_2_-*), 3.05–2.97 (m, 2H, *-CH_2_-*), 2.91 (d, *J* = 1.3 Hz, 3H, *-CH_3_*), 2.61 (s, 3H, *-CH_3_*), 2.17–2.04 (m, 2H, *-CH_2_-*). ^13^C NMR (101 MHz, Deuterium Oxide) δ 160.1, 147.8, 132.7, 131.4, 117.4, 117.2, 110.9, 55.8, 53.6, 48.2, 45.4, 41.1, 32.7, 20.8. HRMS (ESI) m/z [M+H]^+^ Calcd for C_14_H_23_N_6_O^+^: 291.19279. Found: 291.19267.

*N*,*N*’-Dimethyl-*N*’-(1-(3-trifluoromethoxyphenyl)-1H-tetrazol-5-yl-methyl)-1,3-propylenediamine hydrochloride (**10e**)

Colorless oil. Yield: 92%. ^1^H NMR (400 MHz, Deuterium Oxide) δ 7.70–7.64 (m, 1H, *-ArH*), 7.58–7.52 (m, 2H, *-ArH*), 7.48 (ddd, *J* = 7.9, 2.0, 1.0 Hz, 1H, *-ArH*), 4.71 (s, 2H, *-CH_2_-*), 3.26–3.20 (m, 2H, *-CH_2_-*), 3.01–2.95 (m, 2H, *-CH_2_-*), 2.83 (s, 3H, *-CH_3_*), 2.60 (s, 3H, *-CH_3_*), 2.10–2.01 (m, 2H, *-CH_2_-*). ^13^C NMR (101 MHz, Deuterium Oxide) δ 148.2, 132.8, 132.0, 124.2, 123.5, 118.1, 53.7, 48.5, 45.5, 41.1, 32.7, 20.9. HRMS (ESI) m/z [M+H]^+^ Calcd for C_14_H_20_F_3_N_6_O^+^: 345.16452. Found: 345.16431.

#### 3.2.2. Synthesis of **16a**–**e**

General procedure for the preparation of compounds **11c**–**d**.

To a solution of compound **4c**–**d** (2.00 g, 8.06 mmol) in dry dichloromethane was added BBr_3_ (16.12 mL, 1 M solution in CH_2_Cl_2_, 16.12 mmol) dropwise under argon at −78 °C and then the reaction mixture was warmed to room temperature and stirred for 12h. The mixture was washed three times with water, the organic layer was dried over Na_2_SO_4_, and the solvent removed under reduced pressure. The solid residue was purified by column chromatography on silica gel using EtOAc/petroleum as eluent to give **11c**–**d**.

Ethyl 1-(4-hydroxylphenyl)-1H-tetrazole-5-carboxylate (**11c**)

White solid. Yield: 67%. MP: 136.4–137.0 °C. ^1^H NMR (400 MHz, Chloroform-*d*) δ 7.43–7.37 (m, 2H, *-ArH*), 7.06–7.00 (m, 2H, *-ArH*), 4.47 (q, *J* = 7.1 Hz, 2H, *-CH_2_-*), 1.41 (t, *J* = 7.2 Hz, 3H, *-CH_3_*).

Ethyl 1-(3-hydroxylphenyl)-1H-tetrazole-5-carboxylate (**11d**)

White solid. Yield: 67%. MP: 160.4–161.3 °C. ^1^H NMR (400 MHz, DMSO-*d*_6_) δ 10.12 (s, 1H, *-OH)*, 7.39 (t, *J* = 8.0 Hz, 1H, *-ArH*), 7.09 (ddd, *J* = 7.9, 2.1, 1.0 Hz, 1H, *-ArH*), 7.06 (t, *J* = 2.1 Hz, 1H, *-ArH*), 7.02 (ddd, *J* = 8.2, 2.4, 1.0 Hz, 1H, *-ArH*), 4.33 (q, *J* = 7.1 Hz, 2H, *-CH_2_-*), 1.21 (t, *J* = 7.1 Hz, 3H, *-CH_3_*).

General procedure for the preparation of compounds **12a**–**b**.

To a solution of **11c**–**d** (0.60 g, 2.56 mmol) in ethyl acetate was added triethylamine (0.71 mL, 5.12 mmol), methanesulfonyl chloride (0.26 mL, 3.33mmol) at 0 °C and stirred for 10 min. The reaction mixture was quenched with water and extracted with ethyl acetate. The organic layer was dried over Na_2_SO_4_, and the solvent removed under reduced pressure without further purification to give **12a**–**b**.

Ethyl 1-(4-methylsulfonylphenyl)-1H-tetrazole-5-carboxylate (**12a**)

White solid, Yield: 95%. MP: 135.7–137.8 °C. ^1^H NMR (400 MHz, Chloroform-*d*) δ 7.65–7.61 (m, 2H, *-ArH*), 7.57–7.51 (m, 2H, *-ArH*), 4.49 (q, *J* = 7.2 Hz, 2H, *-CH_2_-*), 3.28 (s, 3H, *-SO_3_CH_3_*), 1.42 (t, *J* = 7.1 Hz, 3H, *-CH_3_*).

Ethyl 1-(3-methylsulfonylphenyl)-1H-tetrazole-5-carboxylate (**12b**)

Yellow oil, Yield: 95%. ^1^H NMR (400 MHz, DMSO-*d*_6_) δ 7.91–7.87 (m, 1H, *-ArH*), 7.79–7.72 (m, 2H, *-ArH*), 7.66 (dt, *J* = 6.9, 2.3 Hz, 1H, *-ArH*), 4.32 (q, *J* = 7.1 Hz, 2H, *-CH_2_-*), 3.47 (s, 3H, *-SO_3_CH_3_*), 1.20 (t, *J* = 7.1 Hz, 3H, *-CH_3_*).

General procedure for the preparation of compounds **13a**–**b**.

To a solution of **11c**–**d** (0.40 g, 1.71 mmol) in dry DMF was added K_2_CO_3_ (0.28 g, 2.05 mmol), 2,2,2-trifluoroethyltrifluoromethanesulfonate (0.27 mL, 1.88 mmol) at room temperature and stirred for 3h. The mixture was quenched with water and extracted with ethyl acetate. The organic layer was dried over Na_2_SO_4_, and the solvent removed under reduced pressure without further purification to give **13a**–**b**.

Ethyl 1-(4-(2,2,2-trifluoroethoxy)phenyl)-1H-tetrazole-5-carboxylate (**13a**)

White solid, Yield: 94%. MP: 119.2–120.0 °C. ^1^H NMR (400 MHz, Chloroform-*d*) δ 7.53–7.48 (m, 2H, *-ArH*), 7.16–7.12 (m, 2H, *-ArH*), 4.47 (qd, *J* = 7.5, 4.4 Hz, 4H, *-CH_2_CF_3_, -CH_2_-*), 1.42 (t, *J* = 7.2 Hz, 3H, *-CH_3_*).

Ethyl 1-(3-(2,2,2-trifluoroethoxy)phenyl)-1H-tetrazole-5-carboxylate (**13b**)

White solid, Yield: 94%. MP: 77.0–77.6 °C. ^1^H NMR (400 MHz, DMSO-*d*_6_) δ 7.59 (t, *J* = 8.2 Hz, 1H, *-ArH*), 7.53 (t, *J* = 2.2 Hz, 1H, *-ArH*), 7.38 (dddd, *J* = 9.3, 8.4, 2.3, 1.0 Hz, 2H, *-ArH*), 4.86 (q, *J* = 8.8 Hz, 2H, *-CH_2_CF_3_*), 4.33 (q, *J* = 7.1 Hz, 2H, *-CH_2_-*), 1.20 (t, *J* = 7.1 Hz, 3H, *-CH_3_*).

General procedure for the preparation of compounds **14a**–**d**.

To a solution of **12a**–**b** or **13a**–**b** (1.0 eq) in anhydrous dichloromethane (1.8 mL/mmol) was added diisobutyl aluminium hydride (1.0 M in hexanes, 2.0 eq) dropwise at −78 °C and the reaction was stirred for 30 min. The mixture was quenched with methanol, concentrated and extracted with ethyl acetate. The combined organic layers were washed with water, 1 M HCl and dried over anhydrous Na_2_SO4, filtered and concentrated. The residue was purified by column chromatography on silica gel using EtOAc/petroleum as eluent to give **14a**–**d**.

1-(4-Methylsulfonylphenyl)-1H-tetrazole-5-carbaldehyde (**14a**)

Yellow solid. Yield: 78%. MP: 146.7–147.0 °C. ^1^H NMR (400 MHz, DMSO-*d*_6_) δ 10.10 (s, 1H, *-CHO*), 7.94–7.88 (m, 2H, *-ArH*), 7.68–7.63 (m, 2H, *-ArH*), 3.52 (s, 3H, *-SO_3_CH_3_*).

1-(3-Methylsulfonylphenyl)-1H-tetrazole-5-carbaldehyde (**14b**)

Yellow oil. Yield: 75%. ^1^H NMR (400 MHz, DMSO-*d*_6_) δ 10.09 (s, 1H, *-CHO*), 7.87 (t, *J* = 2.1 Hz, 1H, *-ArH*), 7.77–7.72 (m, 2H, *-ArH*), 7.61 (ddd, *J* = 8.0, 2.3, 1.2 Hz, 1H, *-ArH*), 3.49 (s, 3H, *-SO_3_CH_3_*).

1-(4-(2,2,2-Trifluoroethoxy)phenyl)-1H-tetrazole-5-carbaldehyde (**14c**)

Yellow oil. Yield: 76%. ^1^H NMR (400 MHz, DMSO-*d*_6_) δ 10.06 (s, 1H, *-CHO*), 7.76–7.72 (m, 2H, *-ArH*), 7.33–7.30 (m, 2H, *-ArH*), 4.92 (q, *J* = 8.9, 7.1 Hz, 2H, *-CH_2_CF_3_*).

1-(3-(2,2,2-Trifluoroethoxy)phenyl)-1H-tetrazole-5-carbaldehyde (**14d**)

White solid. Yield: 75%. MP: 101.5–102.0 °C. ^1^H NMR (400 MHz, DMSO-*d*_6_) δ 10.08 (s, 1H, *-CHO*), 7.63 (t, *J* = 8.2 Hz, 1H, *-ArH*), 7.49 (t, *J* = 2.3 Hz, 1H, *-ArH*), 7.42 (ddd, *J* = 5.5, 2.0, 0.9 Hz, 1H, *-ArH*), 7.41–7.39 (m, 1H, *-ArH*), 4.87 (q, *J* = 8.8, 1.3 Hz, 2H, *-CH_2_CF_3_*).

General procedure for the preparation of compounds **15a**–**e**.

To a solution of **14a**–**d** (1.0 eq) in 1,2-Dichloroethane (8.5 mL/mmol) was added sodium triacetoxyborohydride (2.0 eq) and **6a** or **6b** (1.0 eq) and the reaction was stirred for 12 h at room temperature. The reaction mixture was diluted with water and extracted with dichloromethane. The dichloromethane layer was dried over anhydrous Na_2_SO4, filtered and concentrated. The residue was purified by column chromatography on silica gel using EtOAc/petroleum as eluent to give **15a**–**e**.

*N*-tert-Butyloxycarbonyl-*N*-methyl-*N*’-(1-(4-methylsulfonylphenyl)-1H-tetrazol-5-yl-methyl)-*N*’-methyl-ethylenediamine (**15a**)

Colorless oil. Yield: 77%. ^1^H NMR (400 MHz, DMSO-*d*_6_) δ 7.91 (d, *J* = 8.6 Hz, 2H, *-ArH*), 7.65–7.61 (m, 2H, *-ArH*), 3.91 (s, 2H, *-CH_2_-*), 3.50 (s, 3H, *-CH_3_*), 3.13 (t, *J* = 6.5 Hz, 2H, *-CH_2_-*), 2.62 (d, *J* = 15.3 Hz, 3H, *-CH_3_*), 2.48 (d, *J* = 6.5 Hz, 2H, *-CH_2_-*), 2.19 (d, *J* = 6.5 Hz, 3H, *-CH_3_*), 1.33 (d, *J* = 11.8 Hz, 9H, *-C(CH_3_)_3_*).

*N*-tert-Butyloxycarbonyl-*N*-methyl-*N*’-(1-(3-methylsulfonylphenyl)-1H-tetrazol-5-yl-methyl)-*N*’-methyl-ethylenediamine (**15b**)

Colorless oil. Yield: 75%. ^1^H NMR (400 MHz, DMSO-*d*_6_) δ 7.84 (d, *J* = 23.0 Hz, 2H, *-ArH*), 7.77 (t, *J* = 8.1 Hz, 1H, *-ArH*), 7.64 (ddd, *J* = 8.1, 2.4, 1.2 Hz, 1H, *-ArH*), 3.94 (s, 2H, *-CH_2_-*), 3.49 (s, 3H, *-CH_3_*), 3.12 (t, *J* = 6.5 Hz, 2H, *-CH_2_-*), 2.62 (d, *J* = 11.0 Hz, 3H, *-CH_3_*), 2.48 (t, *J* = 6.5 Hz, 2H, *-CH_2_-*), 2.20 (d, *J* = 4.4 Hz, 3H, *-CH_3_*), 1.32 (d, *J* = 8.5 Hz, 9H, *-C(CH_3_)_3_*).

*N*-tert-Butyloxycarbonyl-*N*-methyl-*N*’-(1-(4-(2,2,2-trifluoroethoxy)phenyl)-1H-tetrazol-5-yl-methyl)-*N*’-methyl-ethylenediamine (**15c**)

Colorless oil. Yield: 82%. ^1^H NMR (400 MHz, DMSO-*d*_6_) δ 7.92–7.86 (m, 1H, *-ArH*), 7.77–7.66 (m, 2H, *-ArH*), 7.37–7.33 (m, 1H, *-ArH*), 4.91 (q, *J* = 8.8 Hz, 2H, *-CH_2_-*), 3.85 (s, 2H, *-CH_2_-*), 3.14 (t, *J* = 6.5 Hz, 2H, *-CH_2_-*), 2.64 (d, *J* = 13.8 Hz, 3H, *-CH_3_*), 2.48 (t, *J* = 6.4 Hz, 2H, *-CH_2_-*), 2.18 (s, 3H, *-CH_3_*), 1.33 (d, *J* = 12.7 Hz, 9H, *-C(CH_3_)_3_*).

*N*-tert-Butyloxycarbonyl-*N*-methyl-*N*’-(1-(3-(2,2,2-trifluoroethoxy)phenyl)-1H-tetrazol-5-yl-methyl)-*N*’-methyl-ethylenediamine (**15d**)

Colorless oil. Yield: 80%. ^1^H NMR (400 MHz, DMSO-*d*_6_) δ 7.61 (t, *J* = 8.2 Hz, 1H, *-ArH*), 7.55–7.40 (m, 2H, *-ArH*), 7.34 (dd, *J* = 8.5, 2.5 Hz, 1H, *-ArH*), 4.89 (q, *J* = 8.8 Hz, 2H, *-CH_2_-*), 3.93 (s, 2H, *-CH_2_-*), 3.11 (t, *J* = 6.5 Hz, 2H, *-CH_2_-*), 2.61 (d, *J* = 12.7 Hz, 3H, *-CH_3_*), 2.47 (t, *J* = 6.5 Hz, 2H, *-CH_2_-*), 2.19 (s, 3H, *-CH_3_*), 1.32 (d, *J* = 9.0 Hz, 9H, *-C(CH_3_)_3_*).

*N*-tert-Butyloxycarbonyl-*N*-methyl-*N*’-(1-(3-methylsulfonylphenyl)-1H-tetrazol-5-yl-methyl)-*N*’-methyl-1,3-propylenediamine (**15e**)

Colorless oil. Yield: 75%.^1^H NMR (400 MHz, DMSO-*d*_6_) δ 7.90 (t, *J* = 2.1 Hz, 1H, *-ArH*), 7.82–7.74 (m, 2H, *-ArH*), 7.66–7.62 (m, 1H, *-ArH*), 3.89 (s, 2H, *-CH_2_-*), 3.48 (s, 3H, *-CH_3_*), 2.93 (s, 2H, *-CH_2_-*), 2.67 (d, *J* = 4.1 Hz, 3H, *-CH_3_*), 2.29 (t, *J* = 7.1 Hz, 2H, *-CH_2_-*), 2.10 (s, 3H, *-CH_3_*), 1.46 (s, 2H, *-CH_2_-*), 1.35 (s, 9H, *-C(CH_3_)_3_*).

General procedure for the preparation of compounds **16a**–**e**.

Compounds **15a**–**e** (0.51 mmol) was added to saturated hydrochloric acid ethanol solution (10 mL) at room temperature and stirred for 30 min. The reaction solution was filtered and washed with diethylether (20 mL) to give **16a**–**e**.

*N*,N’-Dimethyl-*N*’-(1-(4-methylsulfonylphenyl)-1H-tetrazol-5-yl-methyl)ethylenediamine hydrochloride (**16a**)

White solid. Yield: 95%. MP: 198.9–199.3 °C. ^1^H NMR (400 MHz, Deuterium Oxide) δ 7.65–7.60 (m, 2H, *-ArH*), 7.56–7.52 (m, 2H, *-ArH*), 4.24 (s, 2H, *-CH_2_-*), 3.32 (s, 3H, -*SO_3_CH_3_*), 3.14 (t, *J* = 6.4 Hz, 2H, *-CH_2_-*), 3.00 (t, *J* = 6.3 Hz, 2H, *-CH_2_-*), 2.58 (s, 3H, *-CH_3_*), 2.41 (s, 3H, *-CH_3_*). ^13^C NMR (101 MHz, Deuterium Oxide) δ 150.2, 150.1, 131.3, 127.1, 124.2, 51.9, 49.4, 44.1, 41.4, 37.1, 33.0. HRMS (ESI) m/z [M+H]^+^ Calcd for C_13_H_21_N_6_O_3_S^+^: 341.13904. Found: 341.13906.

*N*,*N*’-Dimethyl-*N*’-(1-(3-methylsulfonylphenyl)-1H-tetrazol-5-yl-methyl)ethylenediamine hydrochloride (**16b**)

Colorless oil. Yield: 94%. ^1^H NMR (400 MHz, Deuterium Oxide) δ 7.68 (td, *J* = 8.1, 0.7 Hz, 1H, *-ArH*), 7.59 (qd, *J* = 2.5, 0.9 Hz, 2H, *-ArH*), 7.57–7.53 (m, 1H, *-ArH*), 4.32 (s, 2H, *-CH_2_-*), 3.30 (s, 3H, -*SO_3_CH_3_*), 3.20–3.15 (m, 2H, *-CH_2_-*), 3.06 (t, *J* = 6.3 Hz, 2H, *-CH_2_-*), 2.59 (s, 3H, *-CH_3_*), 2.47 (s, 3H, *-CH_3_*). ^13^C NMR (101 MHz, Deuterium Oxide) δ 150.6, 148.9, 133.3, 132.0, 125.2, 124.3, 119.5, 51.9, 49.3, 44.4, 41.3, 37.1, 32.9. HRMS (ESI) m/z [M+H]^+^ Calcd for C_13_H_21_N_6_O_3_S^+^: 341.13904. Found: 341.13892.

*N*,*N*’-Dimethyl-*N*’-(1-(4-(2,2,2-trifluoroethoxy)phenyl)-1H-tetrazol-5-yl-methyl)ethylenediamine hydrochloride (**16c**)

White solid. Yield: 95%. MP: 188.7–190.0 °C. ^1^H NMR (400 MHz, Deuterium Oxide) δ 7.49–7.44 (m, 2H, *-ArH*), 7.21–7.15 (m, 2H, *-ArH*), 4.59 (q, *J* = 8.4 Hz, 2H, *-CH_2_CF_3_*), 4.25 (s, 2H, *-CH_2_-*), 3.16 (t, *J* = 6.4 Hz, 2H, *-CH_2_-*), 3.02 (t, *J* = 6.4 Hz, 2H, *-CH_2_-*), 2.58 (s, 3H, *-CH_3_*), 2.43 (s, 3H, *-CH_3_*). ^13^C NMR (101 MHz, Deuterium Oxide) δ 158.9, 150.1, 126.8, 126.3, 116.3, 65.6, 65.2, 51.8, 49.1, 44.2, 41.3, 33.0. HRMS (ESI) m/z [M+H]^+^ Calcd for C_14_H_20_F_3_N_6_O^+^: 345.16452. Found: 345.16437.

*N*,*N*’-Dimethyl-*N*’-(1-(3-(2,2,2-trifluoroethoxy)phenyl)-1H-tetrazol-5-ylmethyl)ethylenediamine hydrochloride (**16d**)

Colorless oil. Yield: 92%. ^1^H NMR (400 MHz, Deuterium Oxide) δ 7.54 (ddd, *J* = 8.4, 7.6, 0.7 Hz, 1H, *-ArH*), 7.25 (ddd, *J* = 8.6, 2.4, 1.0 Hz, 1H, *-ArH*), 7.20–7.15 (m, 2H, *-ArH*), 4.57 (q, *J* = 8.4 Hz, 2H, *-CH_2_CF_3_*), 4.40 (s, 2H, *-CH_2_-*), 3.23 (td, *J* = 6.2, 1.4 Hz, 2H, *-CH_2_-*), 3.16 (dd, *J* = 7.2, 5.0 Hz, 2H, *-CH_2_-*), 2.61 (s, 3H, *-CH_3_*), 2.54 (s, 3H, *-CH_3_*). ^13^C NMR (101 MHz, Deuterium Oxide) δ 157.9, 131.5, 118.7, 117.6, 112.1, 65.7, 65.3, 51.9, 49.3, 41.2, 32.9. HRMS (ESI) m/z [M+H]^+^ Calcd for C_14_H_20_F_3_N_6_O^+^: 345.16452. Found: 345.16437.

*N*,*N*’-Dimethyl-*N*’-(1-(3-methylsulfonylphenyl)-1H-tetrazol-5-yl-methyl)-1,3-propylenediamine hydrochloride (**16e**)

Colorless oil. Yield: 92%. ^1^H NMR (400 MHz, Deuterium Oxide) δ 7.74–7.67 (m, 1H, *-ArH*), 7.60 (dt, *J* = 7.7, 1.4 Hz, 2H, *-ArH*), 7.57–7.52 (m, 1H, *-ArH*), 4.80 (s, 2H, *-CH_2_-*), 3.31 (s, 5H, *-CH_2_-*, -*SO_3_CH_3_*), 3.02–2.96 (m, 2H, *-CH_2_-*), 2.91 (s, 3H, *-CH_3_*), 2.60 (s, 3H, *-CH_3_*), 2.13–2.04 (m, 2H, *-CH_2_-*). ^13^C NMR (101 MHz, Deuterium Oxide) δ 149.0, 148.0, 132.8, 132.2, 125.5, 124.3, 119.5, 53.7, 48.4, 45.4, 41.2, 37.1, 32.7, 20.8. HRMS (ESI) m/z [M+H]^+^ Calcd for C_14_H_23_N_6_O_3_S^+^: 355.15469. Found: 355.15460.

#### 3.2.3. Synthesis of **18a**–**e** and **20**

General procedure for the preparation of compounds **17a**–**e**.

To a solution of **5a** (1.0 eq) in 1,2-Dichloroethane (8.5 mL/mmol) was added sodium triacetoxyborohydride (2.0 eq) and **6c**–**g** (1.0 eq) and the reaction was stirred for 12 h at room temperature. The reaction mixture was diluted with water and extracted with dichloromethane. The dichloromethane layer was dried over anhydrous Na_2_SO4, filtered and concentrated. The residue was purified by column chromatography on silica gel using EtOAc/petroleum as eluent to give **17a**–**e**.

*N*-tert-Butyloxycarbonyl-*N*’-(1-(4-isopropoxyphenyl)-1H-tetrazol-5-yl-methyl)-*N*’-methyl-ethylenediamine (**17a**)

Colorless oil. Yield: 85%. ^1^H NMR (400 MHz, Chloroform-*d*) δ 7.57–7.43 (m, 2H, *-ArH*), 7.12–7.03 (m, 2H, *-ArH*), 4.70–4.61 (m, 1H, *-OCH(CH_3_)_2_*), 3.31 (s, 2H, *-CH_2_-*), 2.52 (s, 2H, *-CH_2_-*), 1.61 (s, 2H, *-CH_2_-*), 1.45 (s, 9H, *-C(CH_3_)_3_*), 1.42 (d, *J* = 0.6 Hz, 3H, *-CH_3_*), 1.42–1.39 (m, 3H, *-CH_3_*), 1.27 (s, 3H, *-CH_3_*).

*N*-tert-Butyloxycarbonyl-*N*’-(1-(4-isopropoxyphenyl)-1H-tetrazol-5-yl-methyl)-*N*’-methyl-1,3-propylenediamine (**17b**)

Colorless oil. Yield: 80%. ^1^H NMR (400 MHz, DMSO-*d*_6_) δ 7.65–7.59 (m, 2H, *-ArH*), 7.15–7.09 (m, 2H, *-ArH*), 4.77–4.69 (m, 1H, *-OCH(CH_3_)_2_*), 3.78 (s, 2H, *-CH_2_-*), 2.77 (q, *J* = 6.6 Hz, 2H, *-CH_2_-*), 2.32 (t, *J* = 7.1 Hz, 2H, *-CH_2_-*), 2.09 (s, 3H, *-CH_3_*), 1.40 (t, *J* = 6.9 Hz, 2H, *-CH_2_-*), 1.36 (s, 9H, *-C(CH_3_)_3_*), 1.32 (s, 3H, *-CH_3_*), 1.31 (d, *J* = 2.3 Hz, 3H, *-CH_3_*).

*N*-tert-Butyloxycarbonyl-*N*-methyl-*N*’-(1-(4-isopropoxyphenyl)-1H-tetrazol-5-yl-methyl)-ethylenediamine (**17c**)

Colorless oil. Yield: 85%. ^1^H NMR (400 MHz, Chloroform-*d*) δ 7.55–7.49 (m, 2H, *-ArH*), 7.07–7.01 (m, 2H, *-ArH*), 4.64 (p, *J* = 6.1 Hz, 1H, *-OCH(CH_3_)_2_*), 4.09 (s, 2H, *-NH_2_*), 3.39 (d, *J* = 16.0 Hz, 2H, *-CH_2_-*), 2.91 (d, *J* = 17.3 Hz, 2H, *-CH_2_-*), 2.86 (s, 3H, *-CH_3_*), 2.14 (s, 2H, *-CH_2_-*), 1.44 (s, 9H, *-C(CH_3_)_3_*), 1.41 (s, 3H, *-CH_3_*), 1.40 (s, 3H, *-CH_3_*).

*N*-tert-Butyloxycarbonyl-*N*’-(1-(4-isopropoxyphenyl)-1H-tetrazol-5-yl-methyl)-ethylenediamine (**17d**)

Colorless oil. Yield: 85%. ^1^H NMR (400 MHz, Chloroform-*d*) δ 7.48 (d, *J* = 8.5 Hz, 2H, *-ArH*), 7.11–7.01 (m, 2H, *-ArH*), 4.64 (h, *J* = 6.0 Hz, 1H, *-OCH(CH_3_)_2_*), 4.19 (s, 2H, *-CH_2_-*), 3.33 (d, *J* = 5.9 Hz, 2H, *-CH_2_-*), 2.98 (s, 2H, *-CH_2_-*), 1.45 (s, 9H, *-C(CH_3_)_3_*), 1.42 (d, *J* = 1.3 Hz, 3H, *-CH_3_*), 1.40 (d, *J* = 1.4 Hz, 3H, *-CH_3_*).

*N*-tert-Butyloxycarbonylaminoethyl-*N*’-(1-(4-isopropoxyphenyl)-1H-tetrazol-5-yl-methyl)-piperazine (**17e**)

Colorless oil. Yield: 75%. ^1^H NMR (400 MHz, DMSO-*d*_6_) δ 7.65–7.60 (m, 2H, *-ArH*), 7.17–7.10 (m, 2H, *-ArH*), 6.63 (t, *J* = 5.7 Hz, 1H, *-NH-*), 4.74 (hept, *J* = 6.0 Hz, 1H, *-OCH(CH_3_)_2_*), 3.77 (s, 2H, *-CH_2_-*), 2.98 (q, *J* = 6.4 Hz, 2H, *-CH_2_-*), 2.43–2.15 (m, 10H, *-CH_2_-*), 1.37 (s, 9H, *-C(CH_3_)_3_*), 1.32 (s, 3H, *-CH_3_*), 1.31 (s, 3H, *-CH_3_*).

General procedure for the preparation of compounds **18a**–**e**.

Compounds **17a**–**e** (0.51 mmol) was added to saturated hydrochloric acid ethanol solution (10 mL) at room temperature and stirred for 30 min. The reaction solution was filtered and washed with ethanol (20 mL) to give **18a**–**e**.

*N*-(1-(4-Isopropoxyphenyl)-1H-tetrazol-5-yl-methyl)-*N*-methyl-ethylenediamine hydrochloride (**18a**)

White solid. Yield: 95%. MP: 186.5–187.7 °C. 1H NMR (400 MHz, Deuterium Oxide) δ 7.46–7.38 (m, 2H, *-ArH*), 7.13–7.07 (m, 2H, *-ArH*), 4.69–4.65 (m, 1H, *-OCH(CH_3_)_2_*), 4.41 (s, 2H, *-CH_2_-*), 3.14 (td, *J* = 6.3, 1.3 Hz, 2H, *-CH_2_-*), 3.06 (td, *J* = 6.3, 1.3 Hz, 2H, *-CH_2_-*), 2.49 (s, 3H, *-CH_3_*), 1.26 (s, 3H, *-CH_3_*), 1.25 (s,3H, *-CH_3_*). ^13^C NMR (101 MHz, Deuterium Oxide) δ 150.5, 126.7, 125.0, 117.1, 71.9, 52.9, 49.0, 41.2, 35.2, 20.9. HRMS (ESI) m/z [M+H]^+^ Calcd for C_14_H_23_N_6_O^+^: 291.19279. Found: 291.19278.

*N*-(1-(4-Isopropoxyphenyl)-1H-tetrazol-5-yl-methyl)-*N*-methyl-1,3-propylenediamine hydrochloride (**18b**)

Colorless oil. Yield: 92%. ^1^H NMR (400 MHz, Deuterium Oxide) δ 7.42–7.36 (m, 2H, *-ArH*), 7.12–7.07 (m, 2H, *-ArH*), 4.69–4.63 (m, 1H, *-OCH(CH_3_)_2_*), 4.72 (s, 2H, *-CH_2_-*), 3.32–3.24 (m, 2H, *-CH_2_-*), 2.99–2.90 (m, 2H, *-CH_2_-*), 2.86 (s, 3H, *-CH_3_*), 2.04 (tt, *J* = 9.8, 6.6 Hz, 2H, *-CH_2_-*), 1.25 (s, 3H, *-CH_3_*), 1.23 (s, 3H, *-CH_3_*). ^13^C NMR (101 MHz, Deuterium Oxide) δ 159.4, 147.9, 126.7, 124.5, 117.2, 71.9, 53.7, 48.1, 41.1, 36.2, 22.0, 20.9. HRMS (ESI) m/z [M+H]^+^ Calcd for C_15_H_25_N_6_O^+^: 305.20844. Found: 305.20840.

*N*-Methyl-*N*’-(1-(4-isopropoxyphenyl)-1H-tetrazol-5-yl-methyl)-ethylenediamine hydrochloride (**18c**)

White solid. Yield: 92%. MP: 179.7–180.7 °C. ^1^H NMR (400 MHz, Deuterium Oxide) δ 7.43–7.36 (m, 2H, *-ArH*), 7.12–7.06 (m, 2H, *-ArH*), 4.67 (d, *J* = 6.1 Hz, 1H, *-OCH(CH_3_)_2_*), 4.49 (s, 2H, *-CH_2_-*), 3.37 (ddd, *J* = 7.3, 6.1, 1.5 Hz, 2H, *-CH_2_-*), 3.29 (ddd, *J* = 7.7, 6.1, 1.6 Hz, 2H, *-CH_2_-*), 2.65 (s, 3H, *-CH_3_*), 1.25 (s, 3H, *-CH_3_*), 1.24 (s, 3H, *-CH_3_*). ^13^C NMR (101 MHz, Deuterium Oxide) δ 159.2, 149.3, 126.4, 124.7, 117.2, 71.9, 44.5, 43.4, 40.7, 33.1, 20.9. HRMS (ESI) m/z [M+H]^+^ Calcd for C_14_H_23_N_6_O^+^: 291.19279. Found: 291.19269.

*N*-(1-(4-Isopropoxyphenyl)-1H-tetrazol-5-yl-methyl)-ethylenediaminehydrochloride (**18d**)

White solid. Yield: 93%. MP: 183.1–183.9 °C. ^1^H NMR (400 MHz, Deuterium Oxide) δ 7.43–7.38 (m, 2H, *-ArH*), 7.13–7.08 (m, 2H, *-ArH*), 4.69–4.65 (m, 1H, *-OCH(CH_3_)_2_*), 4.52 (s, 2H, *-CH_2_-*), 3.37 (dd, *J* = 7.2, 5.9 Hz, 2H, *-CH_2_-*), 3.26 (ddd, *J* = 7.4, 6.3, 1.1 Hz, 2H, *-CH_2_-*), 1.26 (s, 3H, *-CH_3_*), 1.25 (s, 3H, *-CH_3_*). ^13^C NMR (101 MHz, Deuterium Oxide) δ 159.2, 149.3, 126.4, 124.7, 117.2, 71.9, 44.6, 40.7, 35.5, 20.9. HRMS (ESI) m/z [M+H]^+^ Calcd for C_13_H_21_N_6_O^+^: 277.17714. Found: 291.17709.

*N*-aminoethyl-*N*’-(1-(4-isopropoxyphenyl)-1H-tetrazol-5-yl-methyl)-piperazinehydrochloride (**18e**)

Yellow solid. Yield: 92%. MP: 183.2–183.9 °C. ^1^H NMR (400 MHz, Deuterium Oxide) δ 7.46–7.40 (m, 2H, *-ArH*), 7.11–7.05 (m, 2H, *-ArH*), 4.00 (s, 2H, *-CH_2_-*), 3.35–3.26 (m, 4H, *-CH_2_-*), 3.19 (s, 4H, *-CH_2_-*), 2.82 (s, 4H, *-CH_2_-*), 1.25 (s, 3H, *-CH_3_*), 1.23 (s, 3H, *-CH_3_*). ^13^C NMR (101 MHz, Deuterium Oxide) δ 158.9, 151.7, 126.7, 116.9, 71.9, 52.5, 51.8, 49.3, 48.3, 33.7, 20.9. HRMS (ESI) m/z [M+H]^+^ Calcd for C_17_H_28_N_7_O^+^: 346.23499. Found: 346.23494.

*N*-2,3-bis-(tert-Butoxycarbonyl)guanidino-*N*’-(1-(4-isopropoxyphenyl)-1H-tetrazol-5-yl-methyl)-*N*’-methyl-ethylenediamine (**19**)

To a solution of **18a** (0.26g, 0.80mmol) in dry DMF was added to stirred for 10 min and then 1,3-bis(tert-butoxycarbonyl)-2-methyl-2-thiopseudourea (0.23g, 0.80mmol) was added and stirred for 5 h. The reaction mixture was quenched with water and extracted with ethyl acetate. The organic layer was dried over Na_2_SO_4_, and the solvent removed under reduced pressure. The solid residue thus obtained was purified by column chromatography on silica gel using EtOAc/petroleum (1/2, V/V) as eluent to give **19** as yellow oil (0.22g, 82%). ^1^H NMR (400 MHz, Chloroform-*d*) δ 11.45 (s, 1H, *-NH-*), 8.48 (s, 1H, *-NH-*), 7.65–7.59 (m, 2H, *-ArH*), 7.06–6.99 (m, 2H, *-ArH*), 4.63 (p, *J* = 6.1 Hz, 1H, *-OCH(CH_3_)_2_*), 3.79 (s, 2H, *-CH_2_-*), 3.52 (q, *J* = 5.5 Hz, 2H, *-CH_2_-*), 2.78 (t, *J* = 6.0 Hz, 2H, *-CH_2_-*), 2.39 (s, 3H, *-CH_3_*), 1.53 (s, 3H, *-CH_3_*), 1.52 (s, 9H, *-C(CH_3_)_3_*), 1.41 (s, 9H, *-C(CH_3_)_3_*), 1.39 (s, 3H, *-CH_3_*).

*N*-Guanidino-*N*’-(1-(4-isopropoxyphenyl)-1H-tetrazol-5-yl-methyl)-*N*’-methyl-ethylenediamine hydrochloride (**20**)

Compounds 1**9** (0.55 mmol) was added to saturated hydrochloric acid ethanol solution (10 mL) at room temperature and stirred for 30min. The reaction solution was filtered and washed with ethanol (20 mL) to give **20**. Yellow oil. Yield: 92%. ^1^H NMR (400 MHz, Deuterium Oxide) δ 7.40 (dd, *J* = 8.7, 5.3 Hz, 2H, *-ArH*), 7.10 (dd, *J* = 8.7, 4.7 Hz, 2H, *-ArH*), 4.76 (s, 1H, *-NH-*), 4.66 (d, *J* = 5.0 Hz, 2H, *-CH_2_-*), 3.59 (dt, *J* = 25.7, 6.0 Hz, 2H, *-CH_2_-*), 3.42 (dd, *J* = 14.0, 7.2 Hz, 2H, *-CH_2_-*), 2.87 (d, *J* = 34.7 Hz, 3H, *-NH-*, *-NH_2_*), 1.24 (d, *J* = 6.0 Hz, 6H, *-CH_3_*). ^13^C NMR (101 MHz, Deuterium Oxide) δ 159.4, 148.1, 126.7, 124.5, 117.2, 71.9, 54.5, 48.4, 41.6, 36.3, 29.5, 20.9. HRMS (ESI) m/z [M+H]^+^ Calcd for C_15_H_25_N_8_O^+^: 333.21458. Found: 333.21451.

### 3.3. Radioactive Methylation Assay and MOA Study

The enzyme inhibitory activities were measured by the radioisotope assay in ShangHai Chempartner Co. Ltd. according to the standard protocol. The similar procedure was recently performed in the publication [25] The radioactive methylation assay was performed in 1× assay buffer (modified Tris buffer) system containing enzyme (PRMT1/5), peptide and [^3^H]-SAM solution and compounds on the assay plate. After 250 nL of compound solutions were added to the assay plate, 15 μL PRMT1/5 enzyme solution or 1× assay buffer for negative control was transfer to per well of prepared compound stock plates and the whole system (the final concentration of PRMT1 or PRMT5 was 0.5 nM or 2 nM in the system) incubated for 15 min at room temperature. Then, 10 μL of peptide and (^3^H)-SAM mixed solution was added to each well to start the reaction (the final concentration of (^3^H)-SAM was 0.25 μM in the system) and the reaction incubated for 60 min at room temperature. Afterwards, the reaction was stopped with addition of 5 μL cold SAM solution to per well. Then, 25 μL of volume per well was transferred to Flashplate from assay plate and incubated for 1 h minimum at room temperature. Finally, the Flashplate was washed with dH_2_O and 0.1% Tween-20 three times and then reading plate in Mi crobeta using program ^3^H-Flashplate. The data was analyzed in GraphPad Prism 5 to obtain IC_50_ values.

The MOA study of compound **9a** was performed against PRMT1 with respect to SAM and peptide substrate independently. In brief, the peptide concentration was kept at 100nM (10× Km) and the IC_50_ values of **9a** were determined at different SAM concentrations (0.5, 1, 2, 6, 20 and 60× Km). And then, the SAM concentration was kept at 250 nm (1× Km) and the IC_50_ values of **9a** monitored at different peptide concentration (0.5, 1, 3, 10, 30 and 100× Km). The method is consistent with the procedure described above.

### 3.4. Molecular Modeling

Since the crystal structure of human PRMT1 (hPRMT1) has not been solved, the homology model of hPRMT1 in complex with SAH, which was built by referencing the crystal structure of rat PRMT3 (rPRMT3, PDB 1F3L) as the template according to our previous publication [23], was used for the current molecular modeling study. By overlaying the crystal structures of PRMT6-MS023 [PDB ID 5E8R] with hPRMT1, the position of MS023 was used as the reference to indicate the binding site, and then compound **9a** was docked by using glide module in Schrödinger Release 2017-4 with default settings [33].

The compound **9a** and hPRMT1-SAH complex was then prepared for molecular dynamic simulations. QM calculations were performed by using the B3LYP 6-31G* basis set within Gaussian16 [34] to optimize the molecular geometries of compound **9a** and SAH, and the atom-centered point charges were calculated to fit the electrostatic potential using RESP [35]. The system was explicitly solvated in a truncated octahedral TIP3P water box (12 Å from the complex to avoid periodic artefacts from occurring) by using Amber 16 with the amber ff14SB force field [36] and the Generalized Amber Force field (GAFF) [37]. 11 *K^+^* ions were added to neutralize the charges of the system. The whole system was first optimised by energy minimisations and equilibrations according to our standard protocol [23], and then 100 ns free production molecular dynamic simulation was followed in the NPT ensemble (T = 300 K; P = 1 atm). The long-range electrostatic effects were treated by using Periodic boundary conditions (PBC) and particle-mesh-Ewald method (PME), and the temperature was coupled to an external bath using a weak coupling algorithm [38]. The non-bonded interaction cutoff was set as 8 Å and the bond interactions involving H-atoms were constrained by using the SHAKE algorithm. The time step to solve the Newton’s equations was chosen as 2 fs and the trajectory files were collected every 100 ps for the subsequent analysis.

MM-PBSA and Normal Mode were performed for the binding free energy calculations, based on 300 snapshots collected from the stable trajectory. The binding free energy contribution of each residues was calculated, and only those greater than −0.7 Kcal/mol were recorded for detailed interactions analysis.

### 3.5. Cell Culture

The MDA-MB-231 cell line used in this study was obtained from the American Type Culture Collection (ATCC, Rockville, MD, USA), and was grown in dulbecco’s modified eagle medium (DMEM, Gibco, Thermo Fisher Scientific, Waltham, MA, USA) supplemented with 10% fetal bovine serum (FBS) (Gibco). The cells were incubated at 37 °C and 5% CO_2_.

### 3.6. Cell Viability Assay

The cytotoxicity was determined by the CCK8 assay. Briefly, MDA-MB-231 cells (5 × 10^3^ cells/well) were seeded in 96-well plates in DMEM containing 10% FBS and grew for 24 h. The exponentially growing cells were incubated with various concentrations of compounds for 48h/72h in serum free DMEM at 37 °C (5% CO_2_, 95% humidity). CCK8 reagent (10 μL) was added to each well and incubated for further 2 h, and then the absorbance was analyzed in a multiwell-plate reader (BioTek ELx800) at 450 nm.

### 3.7. Western Blotting

MDA-MB-231 cells were maintained on 6-well plates at the appropriate density. After the attachment, cells were treated with **9a** at indicated concentrations or DMSO control for 48 h. Total cell lysates were separated by SDS-PAGE and transferred onto nitrocellulose membranes. The blots were blocked with 5% nonfat milk for 30 min and the target proteins were probed with appropriate specific antibody overnight at 4 °C, respectively. The blots were washed with TBST for three times and then incubated with anti-rabbit secondary antibody (HRP conjugated) for 1 h. After another three washes, bands were detected in a ChemiScope3400 imaging system using ECL substrate (Millipore, Burlington, MS, USA). Primary antibodies used were as follows: anti-PRMT1 (Cell Signaling Technology no.2449, Danvers, MA, USA), anti-ADMA (Cell Signaling Technology no.13522), anti-SDMA (Cell Signaling Technology no.13222), anti-GAPDH (Cell Signaling Technology no. 5174), anti-H4R3me2a Abcam, ab9231), Wnt3a (Cell Signaling Technology no. 2721s), anti-DDAH (Abcam, ab9231, Cambridge, UK), Wnt5a/b (Cell Signaling Technology no. 2530s), β-catenin (Cell Signaling Technology no. 8480s), MMP9 (Cell Signaling Technology no.1366s). The results were obtained from multiple membranes. No significant differences were observed among loading controls. Therefore, we presented the representative loading control blot image in the figure.

## 4. Conclusions

In summary, through computer aided drug design, we designed and synthesized 22 1,5-substituded tetrazole derivatives. Among them, five compounds (**9a**, **9f**, **16c**, **18a**, **18e**) showed strong inhibitory effects on PRMT1. Compound **9a** was identified as the most potent PRMT1 inhibitor (IC_50_ = 3.5 μM) in the current study, and it showed strong selectivity to PRMT1 (type I PRMT) with respect to PRMT5 (type II PRMT). The MOA assay showed that compound **9a** did not compete with either SAM or peptides, but according to the crystal structure of human PRMT6 with its inhibitor and combining with the molecular dynamic simulation study, it was believed that compound 9a bound to substrate-arginine binding site. By Western blotting, it was confirmed that compound **9a** inhibited the PRMT1 pathway, and the canonical Wnt/β-catenin signaling pathway was down-regulated. The discovery of compound **9a** is likely to prove to be very important for the understanding of PRMT1 function, and is a potential lead compound for future drug design efforts targeting PRMT1.

## Figures and Tables

**Table 1:**
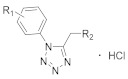
Inhibitory activities of 1,5-substituded tetrazole derivatives against PRMT1 and PRMT5.

Group	Compounds	R_1_	R_2_	% Inhibition of PRMT1 (at 10 μM) ^a^	IC_50_ (μM)	
					PRMT1	PRMT5
I	9a	4-OCH(CH_3_)_2_	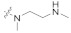	65	3.5	>100
	9b	3-OCH(CH_3_)_2_	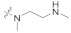	0	-	-
	9c	4-OCH_3_	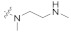	3	-	-
	9d	3-OCH_3_	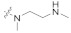	7	-	-
	9f	4-OCF_3_	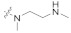	47	23.8	>100
	9e	3-OCF_3_	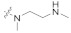	−17	-	-
	16a	4-OSO_2_CH_3_	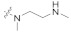	23	-	-
	16b	3-OSO_2_CH_3_	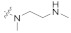	13	-	-
	16c	4-OCH_2_CF_3_	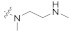	52	19.9	>100
	16d	3-OCH_2_CF_3_	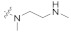	3	-	-
II	10a	4-OCH(CH_3_)_2_	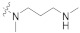	23	-	-
	10b	3-OCH(CH_3_)_2_	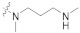	0	-	-
	10c	4-OCH_3_	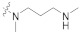	−6	-	-
	10d	3-OCH_3_	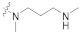	−14	-	-
	10e	3-OCF_3_	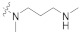	−12	-	-
	16e	3-OSO_2_CH_3_	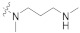	−4	-	-
III	18a	4-OCH(CH_3_)_2_	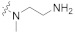	50	10.0	>100
	18b	4-OCH(CH_3_)_2_	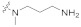	15	-	-
	18c	4-OCH(CH_3_)_2_	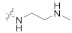	10	-	-
	18d	4-OCH(CH_3_)_2_	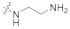	18	-	-
	18e	4-OCH(CH_3_)_2_	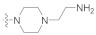	39	29.0	>100
	20	4-OCH(CH_3_)_2_	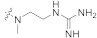	−7	-	-
	SAH				0.49	0.40
	AMI-1				72	-

^a^ Initial inhibition rate was tested at 10 μM for all compounds, and only those with >40% inhibition effects were selected for IC_50_ measurements.

**Table 2:** Calculated energy components, binding free energy (kcal/mol) of compound **9a** in hPRMT1.

Simulations	ΔE_vdw_	ΔE_ele_	ΔG_pb_	ΔG_np_	ΔG_gas_	ΔG_solv_	ΔG_mmpbsa_	TΔS	ΔG_binding_
9a	−38.58 ± 0.22	−257.49 ± 0.70	268.40 ± 0.49	−4.98 ± 0.01	−296.07 ± 0.70	263.43 ± 0.49	−32.64 ± 0.49	−23.00 ± 3.29	−9.64

## Abbreviations

PRMT1	Protein arginine methyltransferase 1
SAM	*S*-adenosyl-L-methionine
mMA	monomethylarginine
SAH	*S*-adenosyl-L-homocysteine
ADMA	Asymmetrical dimethylargine
SDMA	Symmetrical dimethylarginine
DIBAL-H	Diisobutylaluminium hydride
STAB	Sodium triacetoxyborohydride
SAR	Structure-activity relationship
MOA	Mechanism of action
